# *Porphyromonas gingivalis* in Alzheimer’s disease brains: Evidence for disease causation and treatment with small-molecule inhibitors

**DOI:** 10.1126/sciadv.aau3333

**Published:** 2019-01-23

**Authors:** Stephen S. Dominy, Casey Lynch, Florian Ermini, Malgorzata Benedyk, Agata Marczyk, Andrei Konradi, Mai Nguyen, Ursula Haditsch, Debasish Raha, Christina Griffin, Leslie J. Holsinger, Shirin Arastu-Kapur, Samer Kaba, Alexander Lee, Mark I. Ryder, Barbara Potempa, Piotr Mydel, Annelie Hellvard, Karina Adamowicz, Hatice Hasturk, Glenn D. Walker, Eric C. Reynolds, Richard L. M. Faull, Maurice A. Curtis, Mike Dragunow, Jan Potempa

**Affiliations:** 1 Cortexyme, Inc., 269 East Grand Ave., South San Francisco, CA, USA.; 2 Department of Microbiology, Faculty of Biochemistry, Biophysics and Biotechnology, Jagiellonian University, Krakow, Poland.; 3 Malopolska Centre of Biotechnology, Jagiellonian University, Krakow, Poland.; 4 Division of Periodontology, Department of Orofacial Sciences, University of California, San Francisco, San Francisco, CA, USA.; 5 Department of Oral Immunology and Infectious Diseases, University of Louisville School of Dentistry, Louisville, KY, USA.; 6 Broegelman Research Laboratory, Department of Clinical Science, University of Bergen, Bergen, Norway.; 7 The Forsyth Institute, Cambridge, MA, USA.; 8 Harvard University School of Dental Medicine, Boston, MA, USA.; 9 Cooperative Research Centre for Oral Health Science, Melbourne Dental School and the Bio21 Institute of Molecular Science and Biotechnology, University of Melbourne, Melbourne, Victoria, Australia.; 10 Department of Anatomy with Radiology, Centre for Brain Research and NeuroValida, Faculty of Medical and Health Sciences, University of Auckland, Auckland, New Zealand.; 11 Centre for Brain Research and NeuroValida, Faculty of Medical and Health Sciences, University of Auckland, Auckland, New Zealand.; 12 Department of Anatomy and Medical Imaging, Faculty of Medical and Health Sciences, University of Auckland, Auckland, New Zealand.; 13 Department of Pharmacology, Faculty of Medical and Health Sciences, University of Auckland, Auckland, New Zealand.

## Abstract

*Porphyromonas gingivalis*, the keystone pathogen in chronic periodontitis, was identified in the brain of Alzheimer’s disease patients. Toxic proteases from the bacterium called gingipains were also identified in the brain of Alzheimer’s patients, and levels correlated with tau and ubiquitin pathology. Oral *P. gingivalis* infection in mice resulted in brain colonization and increased production of Aβ_1–42_, a component of amyloid plaques. Further, gingipains were neurotoxic in vivo and in vitro, exerting detrimental effects on tau, a protein needed for normal neuronal function. To block this neurotoxicity, we designed and synthesized small-molecule inhibitors targeting gingipains. Gingipain inhibition reduced the bacterial load of an established *P. gingivalis* brain infection, blocked Aβ_1–42_ production, reduced neuroinflammation, and rescued neurons in the hippocampus. These data suggest that gingipain inhibitors could be valuable for treating *P. gingivalis* brain colonization and neurodegeneration in Alzheimer’s disease.

## Teaser

Gingipains from *Porphyromonas gingivalis* drive Alzheimer’s pathology and can be blocked with small-molecule inhibitors.

## INTRODUCTION

Alzheimer’s disease (AD) patients exhibit neuroinflammation consistent with infection, including microglial activation, inflammasome activation, complement activation, and altered cytokine profiles (*1*, *2*). Infectious agents have been found in the brain and postulated to be involved with AD, but robust evidence of causation has not been established (*3*). The recent characterization of amyloid-β (Aβ) as an antimicrobial peptide has renewed interest in identifying a possible infectious cause of AD (*4*–*6*).

Chronic periodontitis (CP) and infection with *Porphyromonas gingivalis—*a keystone pathogen in the development of CP (*7*)*—*have been identified as significant risk factors for developing Aβ plaques, dementia, and AD (*8*–*12*). A prospective observational study of AD patients with active CP reported a notable decline in cognition (Alzheimer’s Disease Assessment Scale—Cognitive and Mini Mental State Examination scales) over a 6-month period compared to AD patients without active CP, raising questions about possible mechanisms underlying these findings (*13*). In *Apoe*^−/−^ mice, oral infection with *P. gingivalis,* but not with two other oral bacteria, results in brain infection and activation of the complement pathway (*14*). In transgenic mice overexpressing mutated human amyloid precursor protein (hAPP-J20), oral infection with *P. gingivalis* impairs cognitive function, increases the deposition of AD-like plaques, and results in alveolar bone loss compared to control hAPP-J20 mice (*15*). *P. gingivalis* lipopolysaccharide has been detected in human AD brains (*16*), promoting the hypothesis that *P. gingivalis* infection of the brain plays a role in AD pathogenesis (*17*).

*P. gingivalis* is mainly found during gingival and periodontal infections; however, it can also be found at low levels in 25% of healthy individuals with no oral disease (*18*). Transient bacteremia of *P. gingivalis* can occur during common activities such as brushing, flossing, and chewing, as well as during dental procedures (*19*), resulting in documented translocation to a variety of tissues including coronary arteries (*20*), placenta (*21*), and liver (*22*). A recent study found that 100% of patients with cardiovascular disease had *P. gingivalis* arterial colonization (*23*).

*P. gingivalis* is an asaccharolytic Gram-negative anaerobic bacterium that produces major virulence factors known as gingipains, which are cysteine proteases consisting of lysine-gingipain (Kgp), arginine-gingipain A (RgpA), and arginine-gingipain B (RgpB). Gingipains are secreted, transported to outer bacterial membrane surfaces, and partially released into the extracellular milieu in soluble and outer membrane vesicle (OMV)–associated forms (*24*, *25*). Kgp and RgpA/B are essential for *P. gingivalis* survival and pathogenicity, playing critical roles in host colonization, inactivation of host defenses, iron and nutrient acquisition, and tissue destruction (*24*, *26*). Gingipains have been shown to mediate the toxicity of *P. gingivalis* in endothelial cells, fibroblasts, and epithelial cells (*27*–*29*). Moreover, because treatment with broad-spectrum antibiotics rarely eradicates *P. gingivalis* and may lead to resistance (*30*), gingipains are implicated as narrow-spectrum virulence targets (*24*, *31*–*33*). Blocking gingipain proteolytic activity with short peptide analogs reduces *P. gingivalis* virulence (*34*).

We hypothesized that *P. gingivalis* infection acts in AD pathogenesis through the secretion of gingipains to promote neuronal damage. We found that gingipain immunoreactivity (IR) in AD brains was significantly greater than in brains of non-AD control individuals. In addition, we identified *P. gingivalis* DNA in AD brains and the cerebrospinal fluid (CSF) of living subjects diagnosed with probable AD, suggesting that CSF *P. gingivalis* DNA may serve as a differential diagnostic marker. We developed and tested potent, selective, brain-penetrant, small-molecule gingipain inhibitors in vivo. Our results indicate that small-molecule inhibition of gingipains has the potential to be disease modifying in AD.

### AD diagnosis correlates with gingipain load in brain

**Fig. 1:**
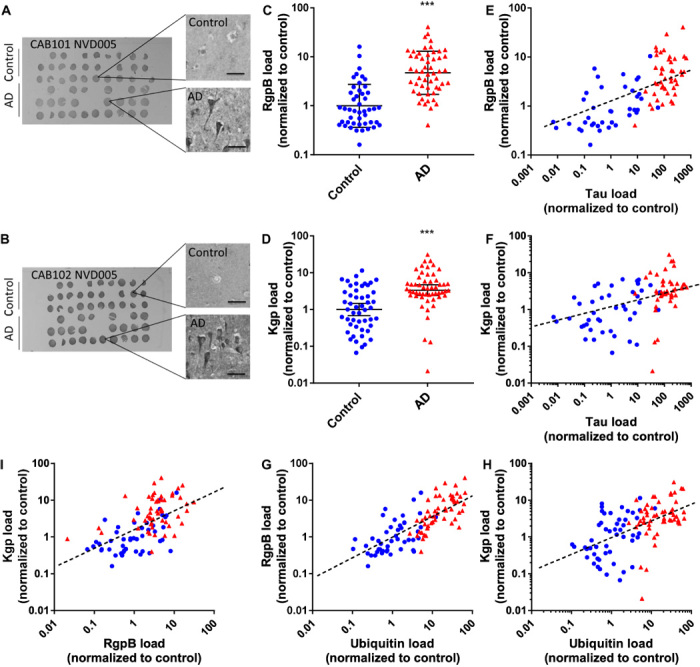
Gingipain IR in brain correlates with AD diagnosis and pathology. (**A** and **B**) Representative TMA NVD005 containing brain tissue cores from the MTG of AD patients and controls probed for RgpB (A) and Kgp (B) with antibodies CAB101 and CAB102, respectively. Higher magnification of representative tissue cores reveals higher neuronal RgpB-IR and Kgp-IR in AD tissue cores than in control cores. (**C**) RgpB-IR and (**D**) Kgp-IR data from TMAs NVD005 and NVD003 show significantly higher load in AD brain compared to controls. Mann-Whitney test, ***P < 0.0001; presented as geometric mean ± 95% confidence interval, *n* = 99 (C) and *n* = 104 (D). (**E** and **F**) Tau load correlates to RgpB load (Spearman *r* = 0.674, *P* < 0.0001, *n* = 84) (E) and Kgp load (Spearman *r* = 0.563, *P* < 0.0001, *n* = 89) (F). Blue, control; red, AD. (**G** and **H**) Ubiquitin load, a marker of AD pathology, correlates to RgpB load (blue, control; red, AD; Spearman *r* = 0.786, *P* < 0.0001, *n* = 99) (G) and Kgp load (Spearman *r* = 0.572, *P* < 0.0001, *n* = 104) (H). (**I**) RgpB load correlates with Kgp load (Spearman *r* = 0.610, *P* < 0.0001, *n* = 99).

Tissue microarrays (TMAs) containing sex- and age-matched brain tissue cores from the middle temporal gyrus (MTG) of both AD patients and neurologically normal individuals were used for immunohistochemical (IHC) studies (tables S1 and S2). Gingipain-specific antibodies, CAB101 and CAB102, targeting RgpB and Kgp, respectively, were used to determine gingipain load in brain tissue cores. Tau load in the TMAs was measured using an antibody (DAKO A0024) that recognizes both nonphosphorylated and phosphorylated tau. RgpB and Kgp exhibited punctate intraneuronal staining in tissue from AD brains (Fig. 1, A and B, respectively). On the basis of threshold analysis (see Materials and Methods), 96% (51 of 53) of AD samples were positive for RgpB and 91% (49 of 54) of AD samples were positive for Kgp. The RgpB load was significantly higher in AD brains than in nondemented control brains (Fig. 1C), and similarly, the Kgp load was significantly higher in AD brains compared to nondemented control brains (Fig. 1D).

We next stained for tau and found a highly significant correlation between RgpB load and tau load (Fig. 1E) and Kgp load and tau load (Fig. 1F). Tau pathology has been shown to correlate with cognitive impairment in AD (*35*). We next stained the TMAs for ubiquitin, a small protein tag that marks damaged proteins for degradation by proteasomes (*36*) and accumulates in both tau tangles and Aβ plaques (*37*). There was a significant correlation between RgpB load and ubiquitin load (Fig. 1G) and Kgp load and ubiquitin load (Fig. 1H) in the TMAs. Of note, in nondemented control tissues, RgpB staining was observed in 39% (18 of 46) of samples and Kgp staining was observed in 52% (26 of 50) of samples. The correlation analyses between the gingipain load and tau load (Fig. 1, E and F) and between the gingipain and ubiquitin load (Fig. 1, G and H) in the nondemented control samples revealed a continuum of gingipain and AD pathology already present in the controls. These findings are consistent with the concept of preclinical AD, i.e., the stage of disease when pathogenesis has begun, but clinical symptoms are not yet present (*38*).

To further validate the gingipain IHC in the TMAs, we performed a correlation analyses between the RgpB load and Kgp load and found a significant correlation between the two different antigens (Fig. 1I). As a further IHC control, brain TMAs from several different non-AD neurological diseases were probed with the CAB101 antibody. RgpB immunostaining on MTG TMAs of Parkinson’s disease, Huntington’s disease, and amyotrophic lateral sclerosis revealed no significant differences compared to controls (fig. S1). In summary, both RgpB and Kgp antigens in brain independently demonstrated a significant correlation with AD diagnosis, tau load, and ubiquitin load.

### RgpB colocalizes with neurons, astrocytes, and pathology in AD hippocampus

**Fig. 2:**
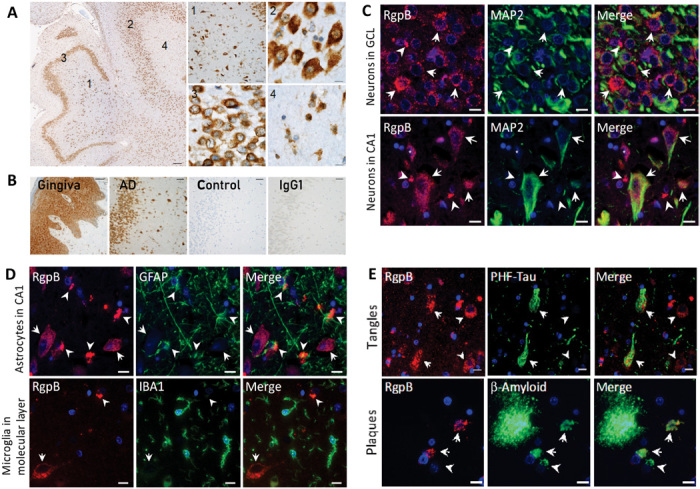
RgpB colocalizes with neurons and pathology in AD hippocampus. (**A**) IHC using RgpB-specific monoclonal antibody 18E6 (representative images from a 63-year-old AD patient). The hippocampus shows abundant intracellular RgpB in the hilus (1), CA3 pyramidal layer (2), granular cell layer (3), and molecular layer (4). High-magnification images from the indicated areas (1 to 4) exhibit a granular staining pattern consistent with *P. gingivalis* intracellular infection. Scale bars, 200 μm (overview), 50 μm (1), and 10 μm (2 to 4). (**B**) AD hippocampus stained with 18E6 (AD) compared to gingival tissue (gingiva) from a patient with periodontal disease as well as a non-AD control and mouse IgG1 control (IgG1) in an adjacent hippocampal section. Scale bars, 50 μm. (**C**) Immunofluorescent colabeling with CAB101 reveals granular intraneuronal staining for RgpB (arrows) in MAP2-positive neurons in both the granular cell layer (GCL) and the pyramidal cell layer (CA1). Scale bars, 10 μm. (**D**) Dense extracellular RgpB-positive aggregates (arrowheads) were closely associated with astrocytes [glial fibrillary acidic protein (GFAP)]. There was no observed association of RgpB with microglia (IBA1). Scale bars, 10 μm. (**E**) RgpB was associated with paired helical filament Tau (PHF-Tau; arrows). RgpB-positive neurons negative for PHF-Tau (arrowheads) were also seen. Intracellular Aβ was often colocalized with RgpB (arrows). In some Aβ-positive cells, RgpB could not be detected (arrowheads). Scale bars, 10 μm.

In AD, the hippocampus is one of the first brain areas to be damaged. Using a different antibody for RgpB than CAB101 (18E6 monoclonal; see Materials and Methods), RgpB-IR was confirmed in neurons of the dentate gyrus and CA3, CA2, and CA1 of AD hippocampus with brightfield microscopy (Fig. 2A). IHC analysis of a series of brains from a university brain bank revealed a similar pattern of staining for RgpB (fig. S2). Using immunofluorescence, RgpB-IR (CAB101) colocalized primarily with neurons [microtubule-associated protein 2 (MAP2)] (Fig. 2C) as well as occasional astrocytes, but not with microglia (Iba1) (Fig. 2D). In addition, RgpB colocalized with pathology including tau tangles and intraneuronal Aβ (Fig. 2E).

### Detection of Kgp in AD cerebral cortex

**Fig. 3:**
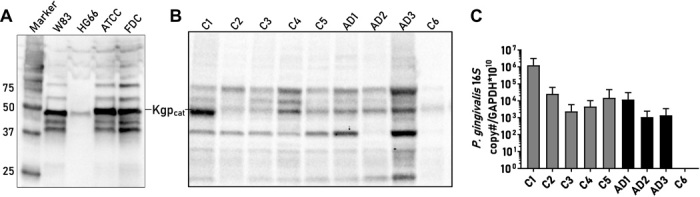
Identification of *P. gingivalis*–specific protein and DNA in cortex from control and AD patients. (**A**) WB with four different strains of *P. gingivalis* and CAB102 detection of typical molecular weight bands for Kgp in bacterial lysates. (**B**) IP using brain lysates from nondemented controls (C1 to C6; ages 75, 54, 63, 45, 37, and 102 years, respectively) and AD patients (AD1 to AD3; ages 83, 90, and 80 years, respectively) using CAB102 with subsequent WB reveals the ~50-kDa Kgp catalytic subunit (Kgp_cat_), along with higher– and lower–molecular weight Kgp species seen in (A). (**C**) qPCR from DNA isolated from the same brain lysates as the protein samples analyzed in (B) shows a positive signal in nondemented control (C1 to C5) and AD (AD1 to AD3) samples. Sample C6 from the 102-year-old nondemented control patient had no detectable qPCR signal in (C) and very faint bands indicating near absence of Kgp (B) (mean with SEM error bars of repeat qPCR runs).

AD is also associated with atrophy of the gray matter of the cerebral cortex. Brain lysates from the cerebral cortex of three AD brains and six nondemented control brains were immunoprecipitated (IP) with CAB102 and run on a Western blot (WB) (Fig. 3B). The CAB102 polyclonal antibody recognizes amino acids 22 to 400 of Kgp, covering the propeptide and the N-terminal region of the catalytic domain (see Materials and Methods). The WB from all three AD brains revealed similar Kgp bands of molecular weights corresponding to the molecular weights of Kgp bands from bacterial lysates from *P. gingivalis* strains W83, ATCC33277, and FDC381 (Fig. 3A). Strain HG66, which contains a mutation affecting the retention of gingipains on its cell surface (*39*), demonstrated only a single Kgp band at the molecular weight of the Kgp catalytic domain (Fig. 3A). The Kgp catalytic domain was identified at the proper molecular weight (*40*) in all of the AD brain samples (Fig. 3B). In addition, five of the six nondemented control brains demonstrated Kgp banding patterns similar to the AD brains (Fig. 3B), consistent with our IHC data demonstrating a continuum of gingipain and AD pathology present in nondemented control brains (Fig. 1, D, F, and H). In the sixth nondemented control brain sample (C6), the Kgp bands were very faint, indicating near absence of Kgp (Fig. 3B).

### Identification of the *P. gingivalis 16S rRNA* and *hmuY* genes in AD cerebral cortex

To further validate the Kgp protein detection data, we performed quantitative polymerase chain reaction (qPCR) analysis on DNA isolated from the same brain tissue used for the Kgp IP and WB analysis. qPCR analysis using *P. gingivalis 16S rRNA* primers revealed the presence of the *P. gingivalis 16S rRNA* gene in the AD brains and five of the six nondemented control brains (Fig. 3C). Control brain C6, which exhibited near absence of Kgp bands in Fig. 3B above, was negative by qPCR for the *P. gingivalis 16S rRNA* gene (Fig. 3C). To further validate the *16S rRNA* qPCR results, we performed PCR analysis using primers for the *hmuY* gene, a gene highly specific for *P. gingivalis* (*41*). All three AD brains and the five nondemented control brains that were positive for the *16S rRNA* gene were also positive for the *hmuY* gene, and sequencing of the *hmuY* PCR products confirmed the presence of *P. gingivalis* in brain DNA (fig. S3). Because we were using a highly sensitive PCR method to detect low copy numbers of *P. gingivalis* DNA (see Materials and Methods), we were concerned that nested amplification of a common Gram-negative bacterium such as *P. gingivalis* in the presence of brain DNA could be creating a false-positive signal. Therefore, as an additional negative control, we used the same nested primer method to attempt to detect another ubiquitous Gram-negative bacterium, *Helicobacter pylori* (see Materials and Methods) (*42*). We tested the three AD brain DNA samples and three of the *P. gingivalis*–positive nondemented brain DNA samples for *H. pylori*. All six brain samples were negative for *H. pylori* using validated qPCR primers and probe (fig. S3D) (*43*), indicating that our *P. gingivalis* PCR results are not likely due to a PCR artifact. In summary, the identification of *P. gingivalis* DNA in AD brains and Kgp-positive nondemented control brains further validates the identification of Kgp in the same brain tissue samples by IP and WB.

### *P. gingivalis* DNA is present in the CSF of clinical AD patients

**Fig. 4:**
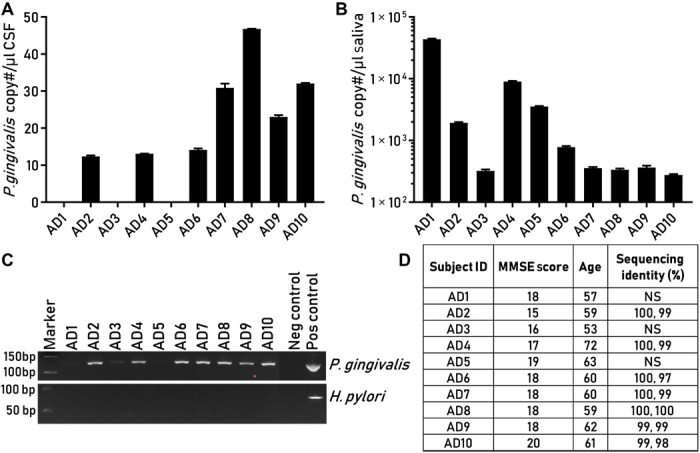
Detection of *P. gingivalis* in CSF and oral biofluids from clinical AD subjects. (**A**) Detection and quantitation of *P. gingivalis* DNA by qPCR in CSF from subjects with probable AD. (**B**) Detection and quantitation of *P. gingivalis* DNA by qPCR from matching saliva samples. (**C**) Top: PCR products detecting *P. gingivalis* from CSF in (A) from all subjects run on agarose gel including negative and positive controls containing a synthetic DNA template. Faint or undetectable PCR products from subjects AD1, AD3, and AD5 were below the limit of quantitation for copy number and not of sufficient quantity for sequence analysis. Bottom: qPCR products from CSF from the same subjects for *H. pylori.* (**D**) Data table includes age and Mini Mental Status Exam (MMSE) score on subjects and sequence identity of PCR products to *P. gingivalis hmuY* DNA sequence. Sequence data are included in fig. S4. NS, not sequenced.

CSF is considered a “window” into brain infection, providing insight into the neuropathogenesis of infectious agents (*44*). Hence, we conducted a prospective pilot study using CSF collected from 10 patients diagnosed with probable AD who had mild to moderate cognitive impairment (Fig. 4D). CSF and matched saliva samples were collected and analyzed for *P. gingivalis* DNA by qPCR detection of the *hmuY* gene (*41*). Positive and negative controls, similar to the standard of care for detection of other brain infections in CSF, were used (*45*, *46*). We were able to detect and quantify copies of the *hmuY* gene by qPCR in CSF in 7 of the 10 clinically diagnosed AD patients, with *P. gingivalis* load ranging from 10 to 50 copies/μl of CSF (Fig. 4A), and the relative fluorescence intensity of the qPCR products on agarose gel was consistent with the qPCR data (Fig. 4C). Sequencing of the endpoint PCR products from CSF confirmed the presence of the *hmuY* gene (fig. S4). We then quantified the *P. gingivalis* load in the matching saliva samples from all 10 patients. All 10 matching saliva samples were positive for *P. gingivalis* by qPCR assay of the *hmuY* gene (Fig. 4B). As with the brain samples noted above, for a PCR-negative control, we analyzed CSF samples for the presence of *H. pylori* using methods with the same sensitivity as for *P. gingivalis*. All of the CSF samples were negative for *H. pylori* (Fig. 4C). The CSF data provide additional evidence for *P. gingivalis* infection in the brain of AD patients.

### Tau is fragmented by gingipains

Because we identified colocalization of gingipain with tau tangles in AD brain (Fig. 2E), we were interested to see whether tau was a target for gingipain proteolysis. Tau truncation and fragmentation have been proposed to play a key role in inducing the formation of insoluble and hyperphosphorylated tau in AD (*47*–*49*). To determine whether gingipains cleave tau in a cell-based system, we used SH-SY5Y cells that express high–molecular weight forms of tau (*50*).

**Fig. 5:**
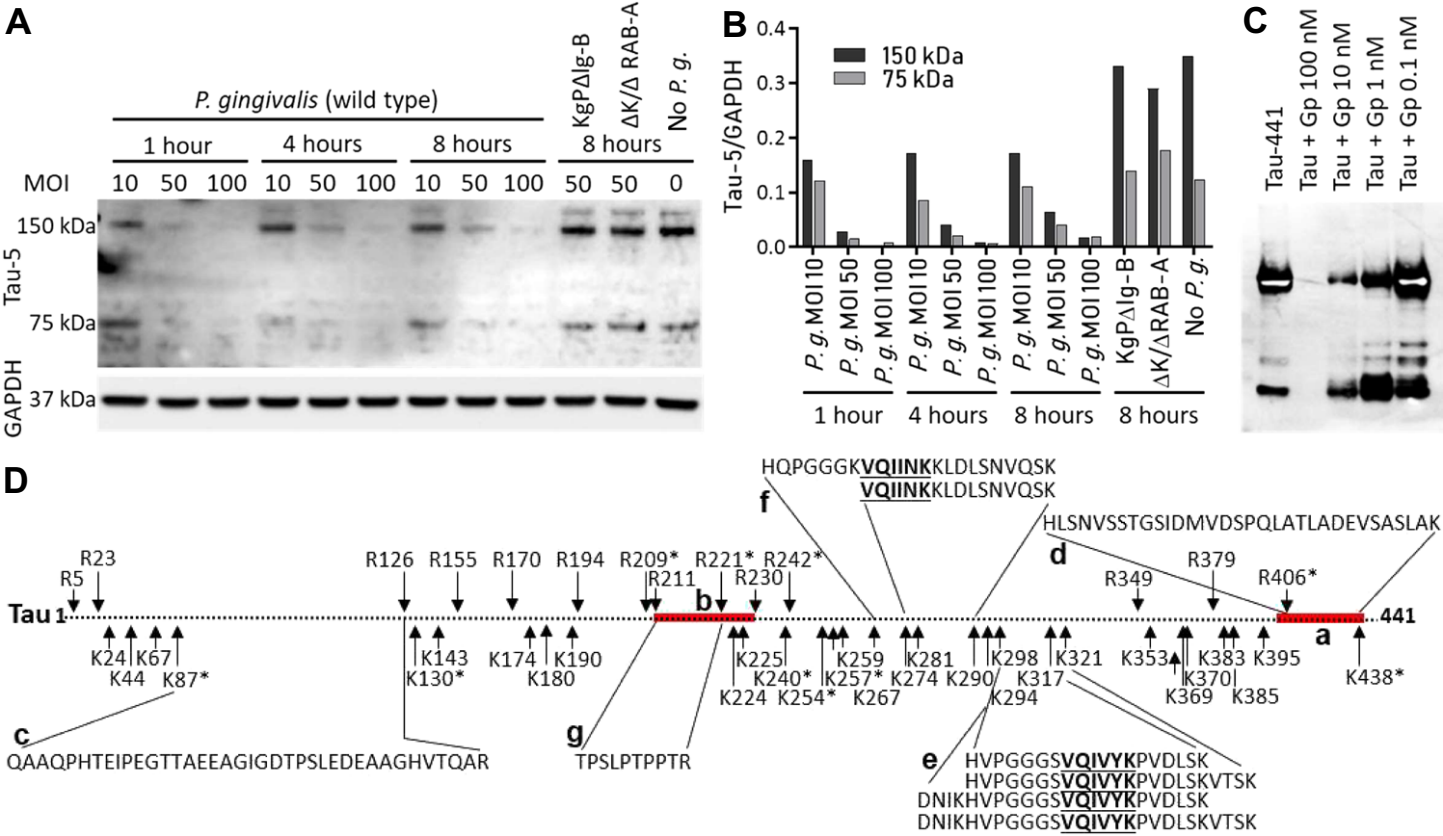
*P. gingivalis* and gingipains fragment tau. (**A**) WB analysis of total soluble tau in SH-SY5Y cells infected with increasing concentrations of wild-type (WT) *P. gingivalis* strain W83 (*P.g.*) and *P. gingivalis* gingipain-deficient mutants either lacking Kgp activity (KgpΔIg-B) or lacking both Kgp and Rgp activity (ΔK/ΔRAB-A)*.* Uninfected SH-SY5Y cells (No *P.g.*) were used as a negative control. Glyceraldehyde-phosphate dehydrogenase (GAPDH) was used as a loading control. Total tau was monitored with the monoclonal antibody Tau-5 at 1, 4, and 8 hours after infection. (**B**) Densitometry analysis of the total tau WB images. (**C**) WB analysis of rtau-441 incubated with purified Kgp and RgpB catalytic domains combined (Gp) at various concentrations for 1 hour at 37°C. The blot was probed with tau monoclonal antibody T46. (**D**) Gingipain cleavage sites in rtau-441 deduced from peptide fragments identified by MS for rtau-441 incubated with 1 or 10 nM gingipains. (a) T46 antibody epitope (red). (b) Tau-5 antibody epitope (red). (c) N-terminal tau fragment. (d) C-terminal tau fragment. (e) Kgp-generated tau fragments containing the VQIVYK sequence. (f) Kgp-generated fragments containing the VQIINK sequence. (g) An RgpB-generated tau fragment. *Cleavage sites identified at 1 nM gingipains.

Using the Tau-5 antibody as a probe, SH-SY5Y cells infected with three different concentrations of *P. gingivalis* were examined at three different time points. The results showed a dose-dependent loss of soluble total tau within 1 hour of infection compared to uninfected cells, while cells infected with *P. gingivalis* gingipain–defective mutants showed soluble tau levels similar to uninfected cells, indicating that gingipains were responsible for the loss of the Tau-5 epitope (Fig. 5, A and B).

To characterize gingipain cleavage sites within tau, we incubated recombinant tau-441 with purified protein containing the catalytic domains of both Kgp and RgpB in combination and identified tau cleavage fragments by mass spectrometry (MS). Following exposure to 1 nM purified gingipains, we identified tau fragments covering 23% of the tau-441 amino acid sequence; at 10 nM gingipains, tau fragments were generated covering 85% of the tau sequence (Fig. 5D and table S3)*.* From the identified tau fragments, we were able to deduce 14 RgpB cleavage sites and 30 Kgp cleavage sites within the tau-441 protein (Fig. 5D). Most of the Kgp cleavage sites (21 of 30) were located C-terminal to position 222 in the tau protein. For RgpB, the majority of cleavage sites (9 of 14) were located N-terminal to position 222. Within the Tau-5 antibody epitope, which spans residues 210 to 230 in tau-441, we identified two RgpB cleavage sites and two Kgp cleavage sites (Fig. 5D, b). Thus, gingipain cleavages within the Tau-5 antibody epitope were the likely cause of the loss of the Tau-5 antibody signal and therefore tau protein detection after SH-SY5Y cells were infected with *P. gingivalis*.

We identified a mid-domain, RgpB-generated tau peptide fragment, TPSLPTPPTR (residues 212 to 221), which is part of the Tau-5 epitope (Fig. 5D, g). This tau peptide is common to all tau isoforms and has been used as an analyte to measure tau levels in CSF (*51*) and determine the turnover rate of tau in the human central nervous system (CNS) (*52*). The TPSLPTPPTR fragment has been reported to be increased 1.7-fold in AD CSF compared to non-AD CSF (*51*).

Kgp generated four unique tau peptide fragments containing the hexapeptide sequence VQIVYK (Fig. 5D, e) and two unique tau peptide fragments containing the VQIINK sequence (Fig. 5D, f). Tau fragments containing these hexapeptide motifs have been shown to be involved in tau tangle formation by nucleating paired helical filaments (PHFs) from full-length tau (*53*, *54*).

### Small-molecule gingipain inhibitors are neuroprotective

**Fig. 6:**
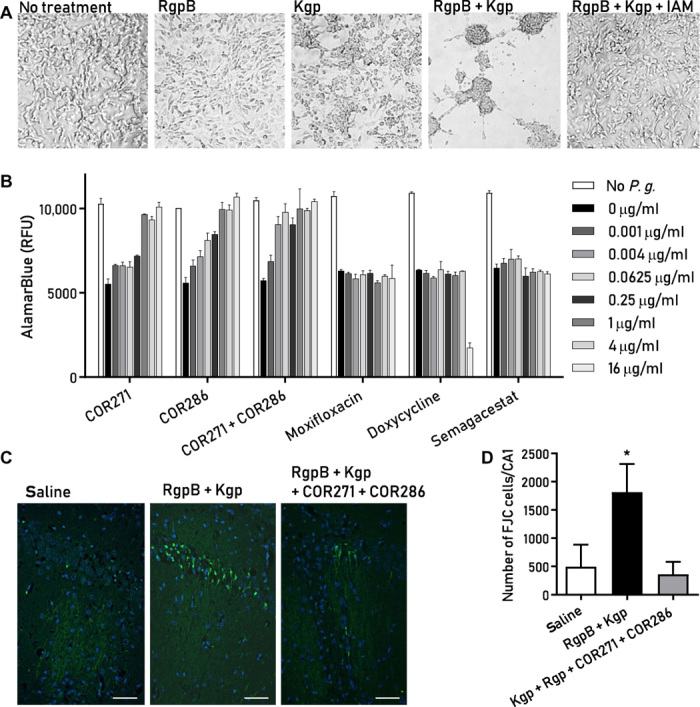
Small-molecule gingipain inhibitors protect neuronal cells against *P. gingivalis*– and gingipain-induced toxicity in vitro and in vivo. (**A**) Differentiated SH-SY5Y neuroblastoma cells demonstrate cell aggregation after exposure to RgpB (10 μg/ml), Kgp (10 μg/ml), or both for 24 hours. The nonselective cysteine protease inhibitor iodoacetamide (IAM) blocks the gingipain-induced cell aggregation. (**B**) AlamarBlue viability assay shows that *P. gingivalis* (*P.g.*) is toxic to SH-SY5Y cells (MOI of 400) and that the small-molecule Kgp inhibitor COR271 and the RgpB inhibitor COR286 provide dose-dependent protection. The broad-spectrum antibiotics moxifloxacin and doxycycline and the γ-secretase inhibitor semagacestat did not inhibit the cytotoxic effect of *P. gingivalis*. (**C**) Fluoro-Jade C (FJC) staining (green) in pyramidal neurons of the CA1 region of the mouse hippocampus indicates neurodegeneration after stereotactic injection of gingipains. Counterstain with 4′,6-diamidino-2-phenylindole (DAPI) (blue). Scale bars, 50 μm. (**D**) The total number of FJC-positive cells was determined from serial section through the entire hippocampus. Results demonstrate a significant neuroprotective effect of gingipain inhibitors COR271 + COR286 after acute gingipain exposure in the hippocampus (*P < 0.05, *n* = 14). All graphs show the mean with SEM error bars.

To determine whether gingipains are toxic to neurons in vitro, we exposed differentiated SH-SY5Y cells to either RgpB or Kgp for 24 hours. Combined application of RgpB and Kgp significantly increased cell aggregation (Fig. 6A). Pretreatment of gingipains with iodoacetamide, an irreversible cysteine protease inhibitor, prevented gingipain-induced aggregation, indicating that the proteolytic activity of the gingipains was responsible for the morphological changes (Fig. 6A).

On the basis of the cytotoxic activity of gingipains from *P. gingivalis*, their presence in AD brain, and their critical role in bacterial survival and virulence, we developed a library of potent and selective reversible and irreversible small-molecule gingipain inhibitors. COR286 and COR271 are irreversible inhibitors of arginine-specific (RgpA and RgpB) and lysine-specific (Kgp) gingipains, respectively, both with a median inhibitory concentration (IC_50_) of <50 pM. COR119 is a reversible covalent Kgp inhibitor with an IC_50_ of 10 nM.

To quantify protective effects of gingipain inhibitors, we infected SH-SY5Y cells with the W83 strain of *P. gingivalis* at a multiplicity of infection (MOI) of 400 for 48 hours, producing approximately 50% cell death (Fig. 6B). COR286 and COR271 were both effective in blocking *P. gingivalis*–induced cell death in a concentration-dependent manner (Fig. 6B). Broad-spectrum antibiotics, moxifloxacin and doxycycline, even at concentrations that reduce bacterial survival in vitro (*55*), did not protect the cells. We also tested the γ-secretase inhibitor semagacestat (LY450139), which blocks the formation of Aβ_1–42_ (*56*), to determine whether bacterial toxicity was mediated by *P. gingivalis*–induced Aβ production, but it had no protective effect (Fig. 6B).

We next assessed whether gingipains are neurotoxic in vivo and whether inhibitors can penetrate the brain and prevent gingipain neurotoxicity. Eight-week-old BALB/c mice were given a single administration of gingipain inhibitors via a combination of COR271 by oral gavage and COR286 subcutaneously, or both vehicles. Stereotactic injection of a combination of Kgp and RgpB into the hippocampus was performed 1.5 hours later. Seven days later, brains were analyzed for neurodegeneration. Mice injected with gingipains had a significantly greater number of degenerating neurons than saline-injected mice, but the neurodegeneration could be blocked by pretreatment with a combination of gingipain inhibitors COR286 and COR271 (Fig. 6, C and D).

### Oral infection of mice with *P. gingivalis* results in brain infection and induction of Aβ_1–42_

**Fig. 7:**
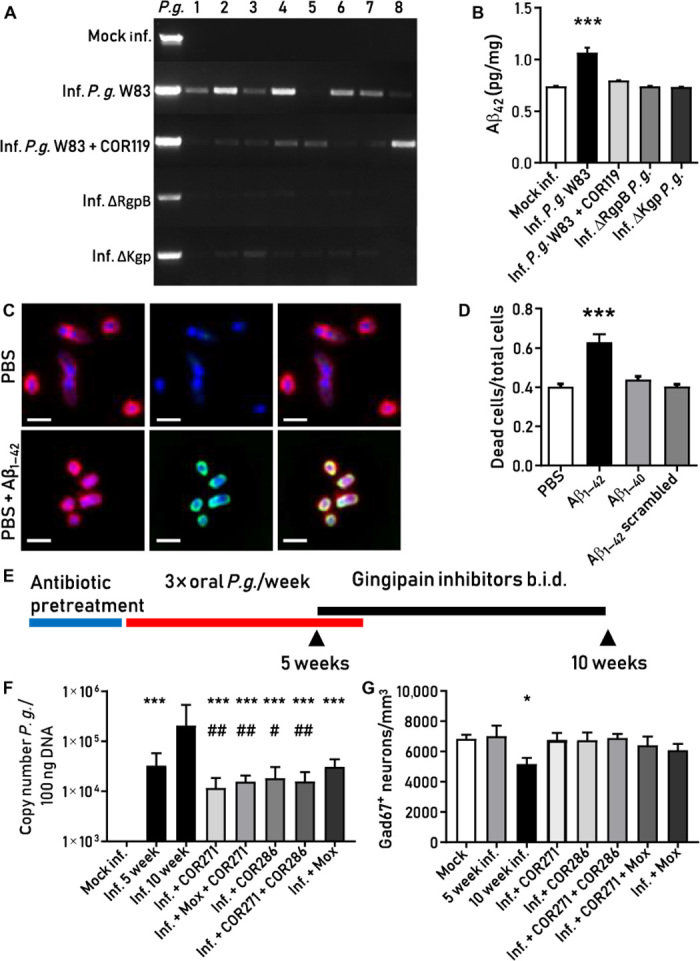
*P. gingivalis* invasion of the brain induces an Aβ_1–42_ response that is blocked by gingipain inhibition in mice. (**A**) *P. gingivalis* PCR product in mouse brains after oral infection with *P. gingivalis* W83, with or without treatment with the Kgp inhibitor COR119, or infection with gingipain knockout strain ΔRgpB or ΔKgp. Lanes 1 to 8 represent individual experimental animals. In the first lane (*P.g.*), *P. gingivalis* W83 was used as a positive control. (**B**) *P. gingivalis* W83–infected mice, but not COR119-treated mice or mice infected with gingipain knockouts, had significantly higher Aβ_1–42_ levels compared to mock-infected mice (***P < 0.001, *n* = 40). (**C**) RgpB-IR (red) colocalized with Aβ_1–42_-IR (green) on the surface of *P. gingivalis* (**D**) Aβ_1–42_, but not Aβ_1–40_ or Aβ_1–42_ scrambled, decreased viability of *P. gingivalis* (***P < 0.001, *n* = 12). (**E**) Study design to quantitate the effect of gingipain inhibitors on brain *P. gingivalis* load. (**F**) qPCR results showed a substantial *P. gingivalis* copy number in the brain at 5 weeks, increasing 10-fold at 10 weeks (Inf. 10 week). All treatment groups showed a significant decrease in *P. gingivalis* load compared to vehicle-treated Inf. 10 week mice (***P < 0.0001, *n* = 63). Treatment with the Kgp inhibitor COR271 resulted in a 90% reduction of *P. gingivalis* copy number. Comparing treatment groups to baseline infection at the beginning of treatment (Inf. 5 week) showed a significant reduction with COR271 and COR286 (^##^*P* < 0.01, ^#^*P* < 0.05) but not with moxifloxacin. (**G**) The number of Gad67^+^ interneurons in the dentate gyrus of the hippocampus was significantly decreased in the Inf. 10 week group (*P < 0.05, *n* = 120). This decrease was reduced in all treatment groups, with COR271 and COR286 trending to better protection than moxifloxacin. (F) Geometric mean with 95% confidence interval. (B), (D), and (G) show the mean with SEM error bars.

We next wanted to understand whether oral exposure to *P. gingivalis* would result in brain infiltration and induction of the stereotypical AD marker Aβ_1–42_. Aged 44-week-old BALB/c mice were orally infected every other day over 6 weeks with *P. gingivalis* W83, Kgp knockout (ΔKgp) [Δ*kgp* (602–1732) Em^r^] (*57*), or RgpA RgpB double knockout (ΔRgp) (Δ*rgpA rgpB*Δ495-B Cm^r^, Em^r^) (*58*) *P. gingivalis.* One W83-infected arm was administered the Kgp inhibitor COR119 three times per day subcutaneously over days 21 to 42. Endpoint PCR analysis of mouse brains for *P. gingivalis* revealed that the bacteria invaded the brain of all eight mice after oral infection for 6 weeks, and colonization was decreased by gingipain knockout strains or treatment with COR119 (Fig. 7A). Mouse brain Aβ_1–42_ increased significantly after oral infection with *P. gingivalis* compared to mock-infected or COR119-treated mice (Fig. 7B). Mice infected with ΔRgp or ΔKgp strains of *P. gingivalis* had brain Aβ_1–42_ levels no different than mock-infected, indicating that both gingipains were needed, either directly or indirectly, to induce an Aβ_1–42_ response in vivo (Fig. 7B).

### Aβ_1–42_ has antibacterial effects against *P. gingivalis*

The significant Aβ_1–42_ response in the mouse brain to *P. gingivalis* infection is consistent with reports demonstrating that Aβ_1–42_ is an antimicrobial peptide (*4*, *6*). We therefore assayed whether Aβ_1–42_ interacts with *P. gingivalis* and decreases its viability in vitro. After incubation of Aβ_1–42_ with *P. gingivalis*, Aβ_1–42_ colocalized with RgpB on the surface of the bacterium (Fig. 7C). Because Aβ_1–42_ is known to disrupt cellular membranes, we hypothesized that Aβ_1–42_ might disrupt the integrity of the *P. gingivalis* membrane to cause cell death. In a separate experiment, we used an assay that can detect damaged bacterial membranes and quantitate the amount of dead or dying bacterial cells as a result of membrane damage. We found that the proportion of dead and dying *P. gingivalis* bacterium significantly increased after incubation with Aβ_1–42_ when compared to Aβ_1–40_, Aβ_1–42_ scrambled, and phosphate-buffered saline (PBS; Fig. 7D).

### Oral administration of a Kgp inhibitor effectively treats *P. gingivalis* brain infection and prevents loss of hippocampal Gad67^+^ interneurons in vivo

We next wanted to measure the effects of gingipain inhibitors on *P. gingivalis* load in the brain after oral infection of BALB/c mice. Eight-week-old mice were orally infected every other day for 42 days with *P. gingivalis* W83 [10^9^ colony-forming units (CFU)] and were given 28 days of rest before study completion. Treatment, beginning after brain infection was established, was administered during days 36 to 70 (Fig. 7E). *P. gingivalis* DNA was identified by qPCR in the brain of all infected mice at day 35, and copies of the *P. gingivalis* genome significantly increased by day 70 (Fig. 7F). The Kgp inhibitor COR271, which has oral bioavailability and significant CNS penetration, administered orally twice a day significantly reduced bacterial load in the brain compared to the positive control infection arm and in comparison to baseline levels at 5 weeks (Fig. 7F). The RgpB inhibitor COR286, administered subcutaneously, was effective in reducing the brain bacterial load at 10 weeks, but was not as effective as COR271 in reducing the bacterial load below baseline at 5 weeks (Fig. 7F). The broad-spectrum antibiotic moxifloxacin was also beneficial in preventing the increase in brain colonization between day 35 and day 70 but was not effective in reducing the *P. gingivalis* load below the 5-week baseline (Fig. 7F). Combinations of COR271 with moxifloxacin or COR286 did not improve efficacy over COR271 alone (Fig. 7F).

Histological analysis of brains after the completion of the 10-week study revealed a significant loss of Gad67^+^ GABAergic interneurons in the hippocampal dentate gyrus in the *P. gingivalis* infection group compared to mock infection (Fig. 7G). In AD, brain neuroimaging and postmortem studies have shown variable disruption of the hippocampal GABAergic system (*59*). Treatment with the Kgp inhibitor COR271 alone, the RgpB inhibitor COR286 alone, a combination of COR271 and COR286, COR271 plus moxifloxacin, and moxifloxacin alone, all beginning at day 36, reduced loss of the Gad67^+^ interneurons, with moxifloxacin-treated arms trending to decreased protection (Fig. 7G).

### COR388 treatment shows dose-dependent effects on brain *P. gingivalis* infection, Aβ_1–42_, and tumor necrosis factor–α levels in mice

On the basis of the superior performance of the highly potent and specific Kgp inhibitor COR271 in the above in vivo mouse study in clearing *P. gingivalis* brain infection and protecting Gad67^+^ neurons in the hippocampus compared to an Rgp inhibitor and a broad-spectrum antibiotic, we developed the COR271 analog COR388. Similar to COR271, COR388 is a highly potent (picomolar inhibition constant on Kgp) and selective irreversible small-molecule inhibitor of Kgp, with superior oral pharmacokinetic and drug-appropriate properties including significant CNS penetration.

**Fig. 8:**
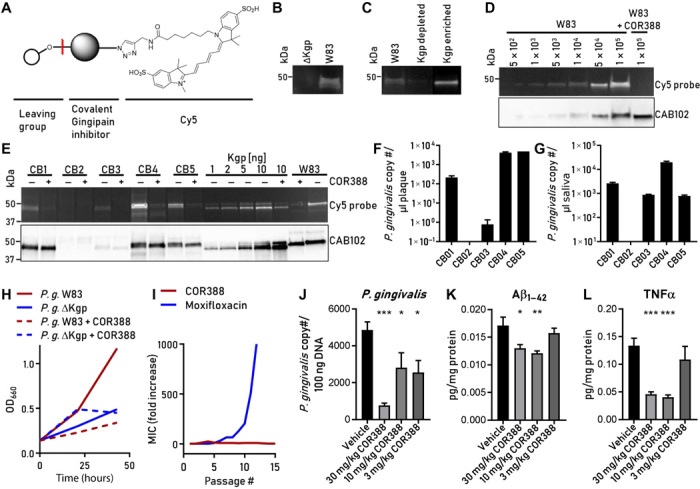
COR388 target engagement and dose-dependent effects on brain *P. gingivalis*, Aβ_1–42_, and TNFα in mice. (**A**) COR553 fluorescent activity probe for Kgp. (**B**) COR553 labeling of Kgp in *P. gingivalis* W83 strain and no labeling in mutant deficient in Kgp (ΔKgp). (**C**) W83 lysates labeled with COR553. Left lane, before immunodepletion; middle lane, after immunodepletion with anti-Kgp–conjugated beads; right lane, after elution from anti-Kgp–conjugated beads. (**D**) W83 strain titrated and labeled with COR553 to determine the limit of bacterial detection. See Results for details. (**E**) Oral plaque samples from human subjects (CB1-5) with periodontal disease were incubated ex vivo with COR553 probe with or without preincubation with COR388. COR553 probe and CAB102 detected Kgp strongly in three subjects (CB1, CB4, and CB5) and weakly in one subject (CB3). COR388 preincubation blocked COR553 probe binding to Kgp. (**F**) qPCR analysis of plaque samples using *hmuY* gene–specific primers identified *P. gingivalis* DNA in samples. (**G**) qPCR analysis of saliva samples. The bar graphs in (F) and (G) show the means and SEMs of three replicates. (**H**) COR388 treatment of W83 culture in defined growth medium reduced growth similarly to a Kgp-deficient strain (ΔKgp) over 43 hours. (**I**) Resistance developed rapidly to moxifloxacin but not COR388 with repeat passaging of bacterial culture. (**J** to **L**) Efficacy of COR388 at three oral doses of 3, 10, and 30 mg/kg twice daily in treating an established *P. gingivalis* brain infection in mice. Reduction of brain tissue levels of *P. gingivalis* (J), Aβ_1–42_ (K), and TNFα (L). The bar graphs show the means with SEM error bars. ***P < 0.001, **P < 0.01, *P < 0.05, *t* test with Dunn’s multiple comparison correction; *n* = 39.

In parallel, we also developed Kgp activity–based probes to characterize COR388 Kgp target engagement in intact *P. gingivalis* bacteria and biological tissue samples. We developed the fluorescent activity probe COR553 by combining a potent, small-molecule irreversible Kgp inhibitor with Cy5 (Fig. 8A) and validated its specificity and potency on Kgp in vitro (Fig. 8, B to D). COR553 bound Kgp present in bacterial cultures of *P. gingivalis* but did not bind a strain deficient in Kgp (Fig. 8B). Preincubation of bacterial lysate with Kgp antibody CAB102 depleted Kgp protein and COR553 binding from the lysate, while CAB102 antibody–bound complexes contain Kgp and COR553 binding, confirming the identity of the COR553 target (Fig. 8C). Preincubation of *P. gingivalis* with 100 nM COR388 before COR553 binding resulted in COR388 engagement with the Kgp active site and a block of COR553 activity probe binding (Fig. 8D). Using the COR553 probe, we demonstrated COR388 Kgp target engagement ex vivo in human oral subgingival plaque samples obtained from patients with periodontal disease (table S4). COR553 labeled Kgp present in plaque in four of the five subjects. Preincubation with COR388 blocked probe binding to the active site, while total Kgp protein was still detected by CAB102 (Fig. 8E). High levels of Kgp in these plaque samples mirrored detection of *P. gingivalis* DNA in the plaque samples (Fig. 8F). These same four subjects also had detectable levels of *P. gingivalis* in saliva (Fig. 8G).

Using a defined growth medium, we showed that COR388 inhibited the growth of *P. gingivalis*, demonstrating that an inhibitor of the Kgp virulence factor involved in generating nutrient amino acids for energy acts as a narrow-spectrum antibiotic (Fig. 8H). To test for the potential for resistance to COR388, *P. gingivalis* was passaged in the presence of COR388 or the broad-spectrum antibiotic moxifloxacin. As shown in Fig. 8I, *P. gingivalis* developed complete resistance to moxifloxacin, with the minimum inhibitory concentration (MIC) increasing over 1000-fold in 12 passages. Resistance to COR388 in two independent assays did not develop in this study. Efficacy of COR388 was tested in vivo to treat an established *P. gingivalis* brain infection in the mouse model described above for COR271 efficacy testing (Fig. 7E). Similar to COR271, oral dosing of COR388 twice daily resulted in dose-dependent efficacy when administered to an established *P. gingivalis* brain infection. Doses of 10 and 30 mg/kg reduced *P. gingivalis* load, Aβ_1–42_, and tumor necrosis factor–α (TNFα) levels in brain tissue compared to those of infected animals treated with vehicle (Fig. 8, J to L). The lowest dose of COR388 at 3 mg/kg showed some reduction of brain *P. gingivalis* load but did not reduce levels of brain Aβ_1–42_ or TNFα (Fig. 8, J to L). Investigational new drug application–enabling studies were completed with COR388, and the compound is currently in clinical studies (ClinicalTrials.gov NCT03331900).

## DISCUSSION

The findings of this study offer evidence that *P. gingivalis* and gingipains in the brain play a central role in the pathogenesis of AD, providing a new conceptual framework for disease treatment. Accordingly, we demonstrate the presence of *P. gingivalis* DNA and gingipain antigens in AD brains and show in vivo that oral administration of small-molecule gingipain inhibitors blocks gingipain-induced neurodegeneration, significantly reduces *P. gingivalis* load in the mouse brain, and significantly decreases the host Aβ_1–42_ response to *P. gingivalis* brain infection.

Our identification of gingipain antigens in the brains of individuals with AD and also with AD pathology but no diagnosis of dementia argues that brain infection with *P. gingivalis* is not a result of poor dental care following the onset of dementia or a consequence of late-stage disease, but is an early event that can explain the pathology found in middle-aged individuals before cognitive decline (*60*). We also demonstrate that *P. gingivalis* bacterial load can be detected in the CSF of clinical AD patients, providing further evidence of *P. gingivalis* infection of the CNS.

The PCR analysis of *P. gingivalis* in the brain and CSF reported here does not differentiate between *P. gingivalis* strains, and future studies are needed to determine what *P. gingivalis* strains are present in the brain and CSF and whether some strains might be more virulent than others in causing disease. In addition, there is one other species of *Porphyromonas* that is known to produce gingipains, *Porphyromonas gulae* (*61*). *P. gulae* is a natural inhabitant of the oral cavity of companion animals such as dogs, and a recent study demonstrated that dogs can transmit *P. gulae* to the oral cavity of their owners (*62*). Research is underway to determine whether *P. gulae* may be contributing to the gingipain load in AD brains.

Evidence from our work reported here lends support to the emerging concept that Aβ is an antimicrobial peptide (*4*–*6*), and mutations (*63*, *64*) contributing to loss of this function could allow more robust infection with *P. gingivalis* and higher risk for disease. In addition, sustained high levels of antimicrobial Aβ driven by chronic *P. gingivalis* infection of the brain may be toxic to host cells, and therefore, reduction of Aβ levels after treatment of the *P. gingivalis* infection should be beneficial. Furthermore, Down syndrome (DS), the most common genetic cause of mental disability, has been used to support Aβ as a therapeutic target because of the notably high prevalence of dementia with Alzheimer-type pathology in DS patients (greater than 50% after the age of 60) and the fact that the amyloid precursor protein gene, which gives rise to Aβ, is present on chromosome 21, which is triplicated in DS (*65*). However, in support of our hypothesis, an aggressive form of periodontitis with rapid progression and onset as early as 6 years of age is associated with DS, but not age-matched normal controls or other mentally handicapped patients of a similar age distribution (*66*). The occurrence of *P. gingivalis* has been found to be significant in the subgingival plaque of DS patients beginning around the age of 5 years when compared to age-matched controls, indicating that *P. gingivalis* abnormally colonizes DS patients in early childhood (*67*). The reason behind DS patients being susceptible to *P. gingivalis* infection at such an early age is unclear but may be due to the immunodeficiency that is associated with DS (*68*). Research is needed to determine whether *P. gingivalis* and gingipains are present in DS CSF and brain.

Although not specifically addressed in this report, once the oral cavity is infected, *P. gingivalis* may access the brain and spread via a number of pathways including (i) infection of monocytes followed by brain recruitment (*69*, *70*), (ii) direct infection and damage to endothelial cells protecting the blood-brain barrier (*28*), and/or (iii) infection and spreading through cranial nerves [e.g., olfactory (*71*) or trigeminal] to the brain. After entering the brain, we suggest that *P. gingivalis* may spread slowly over many years from neuron to neuron along anatomically connected pathways, similar to what has been demonstrated for vascular cell-to-cell transmission of *P. gingivalis (**72**).*

Tau pathology has also been suggested to spread from neuron to neuron (*73*), with a pattern resembling an infectious process. Our data indicate that tau is a target of gingipain proteolysis, and we propose that tau pathology seen in AD brains may be due to the transneuronal spread of *P. gingivalis*, with direct damage of tau by gingipain proteolysis as well as gingipain activation of human proteases that act on tau. Gingipains have been shown to directly cleave procaspase-3 to activate caspase-3 (*74*), a caspase that has been implicated in both tau phosphorylation (*75*) and tau cleavage (*76*). Proteolysis of tau by gingipains would be predicted to increase the turnover rate of tau and trigger a compensatory increase in tau production rate to maintain homeostasis in neurons infected by *P. gingivalis*. Recent research on the kinetics of tau in the human CNS using the tau mid-domain TPSLPTPPTR fragment as a reporter found that the production rate of tau was increased in CSF of subjects with preclinical and clinical AD (*52*). Our data demonstrating that both Kgp and RgpB independently correlate with tau load in AD brains lend support to the hypothesis that gingipains may be a driver of a compensatory increase in tau production. Last, further research is needed to determine whether the gingipain-generated C-terminal tau fragments containing the hexapeptide microtubule-binding domains that we identified in vitro can drive tau filament formation in vivo.

Here, we have not addressed how *P. gingivalis* infection might relate to apolipoprotein E4 (*APOE4*), the greatest genetic risk factor for sporadic AD (*77*). Studies in mice deficient in APOE proteins demonstrated an impaired innate immune response to the bacterial pathogen *Listeria monocytogenes* (*78*), implicating APOE in normal innate immune function in vivo. It was recently reported that human APOE is a target of gingipain proteolysis, and the authors suggested that this mechanism could generate neurotoxic APOE fragments in the AD brain (*79*). We propose that APOE4 may be more susceptible to gingipain cleavage than APOE3 or APOE2 due to the presence of more arginine residues, resulting in decreased innate immune function and the generation of neurotoxic fragments (*80*). The distinct role of APOE in relation to *P. gingivalis* infection and targeting by gingipains remains a focus of future studies.

Our identification of *P. gingivalis* in the CNS underscores the importance of genetic findings linking innate immune response genes to AD susceptibility, including *TREM2* (*81*), *TLR4* (*82*), *CR1* (*83*), and *NLRP3* (*84*). For example, recent studies have highlighted the association between variants of triggering receptor expressed on myeloid cells 2 (*TREM2*) and AD (*81*). *TREM2* encodes a receptor expressed on immune cells such as macrophages and microglia, with heterozygous *TREM2* variants conferring a risk of developing AD similar to one copy of *APOE4* (*81*). TREM2 has been shown to regulate inflammatory responses (*85*) and serve as a phagocytic receptor for bacteria (*86*). TREM1, which shares homology with TREM2, has also been linked to AD amyloid pathology and cognitive decline (*87*). The risk-associated *TREM1* allele was shown to decrease TREM1 surface expression on monocytes (*87*). *P. gingivalis* has been shown to induce *TREM1* gene expression (*88*), and it is therefore possible that carriers of the *TREM1* AD–associated allele have a reduced ability to respond to infection by *P. gingivalis.* In addition, TREM1 is a target for gingipain proteolysis and degradation, with data showing that Rgp can cleave soluble TREM1 from the cell surface and that Kgp can degrade TREM1, actions that could induce chronic inflammation (*88*). Additional research is needed to determine whether TREM2 is involved in the innate immune response to *P. gingivalis* and whether *P. gingivalis* and gingipains have similar effects on the expression and degradation of TREM2 as they do for TREM1. In summary, we propose that genetic polymorphisms of innate immune system genes in essential immune pathways may result in defective clearance of *P. gingivalis* and gingipains from the brain, resulting in chronic, low-level infection and neuroinflammation in susceptible individuals.

With regard to infection-induced neuroinflammation, inflammasomes, multiprotein complexes that act as intracellular innate immune defense systems (*89*), have been shown to be activated in AD brains (*90*). *P. gingivalis* has been shown to modulate inflammasome activity (*91*). Recent research indicates that Aβ plaque formation in AD is connected to the innate immune response through activation of the NLRP3 inflammasome in microglia and release of ASC specks that drive Aβ assembly and deposition (*92*). Notably, *P. gingivalis* was the first microbial pathogen shown to induce ASC aggregation specks in *P. gingivalis*–infected primary human monocytes through activation of the NLRP3 inflammasome (*93*). Inflammasomes act in intracellular innate immune defense against intracellular pathogens by activating interleukin-1β (IL-1β) and IL-18, causing cell death through pyroptosis and thereby eliminating the intracellular niche for pathogen replication (*94*). Furthermore, recent reports have shown that *P. gingivalis* OMVs, nanoscale proteoliposomes that are enriched in gingipains and released into surrounding tissues, are rapidly internalized into mammalian cells (*95*), where they drive NLRP3 inflammasome activation and ASC speck formation and cause cell death through pyroptosis (*96*, *97*). This research suggests that *P. gingivalis* in the human brain, through release of OMVs enriched in gingipains, could drive NLRP3 inflammasome activation, ASC speck aggregation, and subsequent Aβ plaque formation. In addition, recent evidence has shown that the NLRP1 inflammasome in neurons can detect bacterial virulence factors such as proteases by serving as a substrate for the pathogenic enzymes (*98*). We suggest that intraneuronal gingipains may therefore drive neuronal NLRP1 activation, resulting in pyroptosis of neurons and activation of caspase-1, leading to release of the neuroinflammatory interleukins IL-1β and IL-18.

Last, we have shown that broad-spectrum antibiotics do not protect against *P. gingivalis*–induced cell death in vitro, whereas gingipain inhibitors do. We also demonstrated in vivo that an orally administered Kgp inhibitor is more effective than a high-dose subcutaneous broad-spectrum antibiotic in clearing *P. gingivalis* from the brain. It was recently demonstrated that a small-molecule inhibitor of a *Clostridium difficile* cysteine protease virulence factor, TcdB, reduced disease pathology in a mouse model of *C. difficile–*induced colitis but did not reduce the *C. difficile* bacterial load (*99*). In contrast, we report here that small-molecule inhibition of the cysteine protease Kgp reduced not only disease pathology in mouse brain but also *P. gingivalis* bacterial load. The mechanisms underlying the decrease in *P. gingivalis* bacterial load in the brain by Kgp inhibitors are likely due to reduction of Kgp-generated peptide nutrients essential for the growth of this asaccharolytic bacterium (*100*) and blocking of Kgp-dependent heme acquisition that is critical for *P. gingivalis* energy production (*101*, *102*). We have demonstrated that *P. gingivalis* develops rapid resistance to a broad-spectrum antibiotic, moxifloxacin, but not to the Kgp inhibitor COR388. Therefore, with the growing concern about widespread antibiotic resistance (*103*), and severe side effects such as *C. difficile* colitis from broad-spectrum antibiotic use (*104*), an antivirulence factor inhibition approach to treatment of *P. gingivalis* is the most promising path while reducing pressures for resistance.

In conclusion, we have designed an orally bioavailable, brain-penetrant Kgp inhibitor currently being tested in human clinical studies for AD. The present data indicate that treatment with a potent and selective Kgp inhibitor will reduce *P. gingivalis* infection in the brain and slow or prevent further neurodegeneration and accumulation of pathology in AD patients.

## MATERIALS AND METHODS

### Study design

This study was conducted to investigate the prevalence of *P. gingivalis* in the AD brain and to elucidate possible *P. gingivalis*–dependent mechanisms of action for neurodegeneration and AD pathology. In addition, we performed a series of preclinical studies to enable the development of a therapeutic compound against *P. gingivalis*–induced AD. To demonstrate the presence of gingipain antigens in the AD brain, TMAs containing nondemented control and AD brain tissue were used for IHC. To avoid potential bias and subjective elements in assessing the results, stained TMAs were scanned and images were analyzed for gingipain IR using the MetaMorph image analysis program. Evidence for the presence of *P. gingivalis* in the AD brain was further verified by IP, WB, and PCR. To demonstrate the presence of *P. gingivalis* in the CNS of living patients prospectively diagnosed with probable AD, CSF was analyzed for *P. gingivalis* by PCR. The in vitro experiments to demonstrate *P. gingivalis* fragmentation of tau analyzed by WB and MS were designed after detecting the correlation of increased tau load with gingipains in the TMAs and the colocalization of gingipain with tau tangles in human AD brain. To study the efficacy of gingipain inhibitors and neurodegenerative effects of chronic *P. gingivalis* infection in vivo, we developed a mouse model for chronic infection with *P. gingivalis*. The sample size for the mouse model for brain infection with *P. gingivalis* was empirically determined on the basis of effect size and SD. A blinded observer performed quantification of the loss of hippocampal GABAergic neurons after *P. gingivalis* brain infection and the number of degenerating neurons after intrahippocampal injection of gingipains. The efficacy of the top lead compound, COR388, was determined in the brain by qPCR for *P. gingivalis*–specific genes and by enzyme-linked immunosorbent assay (ELISA) for Aβ_1–42_ and TNFα. Animals were assigned to each experimental group with an equal probability of receiving vehicle or treatment.

### Human tissue samples

The human postmortem brain tissue obtained from the Neurological Foundation of New Zealand Human Brain Bank at the University of Auckland was donated to the Brain Bank with family consent, and its use for this study was approved by the University of Auckland Human Participants Ethics Committee. The control cases had no history of neurological abnormalities, and cause of death was unrelated to any neurological condition. Independent pathological analysis confirmed that any amyloid pathology was deemed normal for age in the control cases selected for this study. Pathological analysis was carried out on all AD cases used in this study to determine pathological diagnosis and to assign pathological grades, which, together with a history of dementia, confirmed the diagnosis (tables S1 and S2).

Postmortem tissue samples collected under institutional review board (IRB)–approved protocols were obtained from the University of California Davis Alzheimer’s Disease Center, the University of California San Francisco (UCSF) Neurosurgery Tissue Bank, ProteoGenex (Culver City, CA), and PrecisionMed (Solana Beach, CA). Gingival tissue samples were collected from human volunteers with chronic periodontal disease who provided signed informed consent after the nature and possible consequences of the studies were explained under a University of California at San Francisco IRB–approved protocol (approval no. 11-05608). CSF and saliva samples were collected from human volunteers with a diagnosis of probable AD who provided signed informed consent after the nature and possible consequences of the studies were explained under an IRB-approved protocol obtained from PrecisionMed (Solana Beach, CA). Oral plaque and saliva samples were collected from human volunteers with chronic periodontal disease who provided signed informed consent after the nature and possible consequences of the studies were explained under an IRB-approved protocol obtained from the Forsyth Institute (Cambridge, MA).

### Animals

Specific pathogen–free (SPF) female BALB/c mice were purchased from Envigo (UK) for the oral infection experiments with *P. gingivalis.* Mice were maintained in individually ventilated cages and fed a standard laboratory diet and water ad libitum under SPF conditions within the animal care facility at Faculty of Biochemistry, Biophysics and Biotechnology, the Jagiellonian University, Krakow, Poland. Mice were kept under a 12-hour light/dark cycle at 22° ± 2°C and 60 ± 5 relative humidity. Control and bacterially infected mice were housed in separate cages. To study Aβ_1–42_ levels in the brains of orally infected mice and the efficacy of gingipain inhibitors to decrease *P. gingivalis* infection of the brain, 40 (*n* = 8 per arm) 43- to 44-week-old female BALB/c mice or 100 (*n* = 10 per arm) 8-week-old female BALB/c mice were used, respectively. All the experiments were reviewed and approved by the I Regional Ethics Committee on Animal Experimentation, Krakow, Poland (approval nos. 164/2013 and 116/2016).

To study neurodegeneration after stereotactic injection of gingipains into mouse hippocampus, fifteen 8-week-old male BALB/c mice were purchased from Envigo (USA). Animals were group-housed (*n* = 2 to 4 per cage) in plastic cages. Animals were maintained on a 12/12-hour light/dark cycle with the room temperature (RT) maintained at 22° ± 2°C and approximately 50% humidity and received standard rodent chow and tap water ad libitum in the Brains On-Line, LLC Animal Facility (South San Francisco, CA). Experiments were conducted in accordance with the protocols approved by the Institutional Animal Care and Use Committee of Brains On-Line, LLC (approval no. US16003).

### Antibody production

Polyclonal antibodies CAB101 and CAB102 were produced by GenScript USA Inc. (New Jersey) according to their express immunization protocol. Briefly, immunogens were expressed in a bacterial expression system and used for four consecutive immunizations of four rabbits each. The immunogen sequences expressed are 401 to 736 residues for CAB101 (RgpB; GenBank: BAG33985.1) and 22 to 400 residues for CAB102 (Kgp; GenBank: BAG34247.1). After the last immunization, sera were pooled and antigen affinity–purified. Specific binding was tested on WBs, and nonspecific binding on human histology sections was controlled by coincubation of polyclonal antibodies with their respective immunogens before IHC staining.

### Human TMAs

Human brain TMAs comprised a total of 58 2-mm-diameter core samples, 29 from dementia-free control individuals and 29 from AD cases, each on two arrays (NVD003 and NVD005). Final sample sizes reflect the loss of several samples from the slide during processing (see tables S1 and S2).

### IHC to detect gingipains, tau, and ubiquitin in human TMAs

TMAs were constructed from paraffin-embedded MTG blocks, as described in detail by Narayan *et al.* (*105*). TMA sections were cut at a thickness of 7 μm and were annealed to slides by heating at 60°C for 1 hour. Sections were then dewaxed using xylene immersion in xylene twice (1 hour and 10 min, respectively) and rehydrated using a standard graded ethanol series procedure. For IHC, slides were immersed in sodium citrate antigen retrieval buffer (pH 6), heated at 121°C for 2 hours in 2100 Antigen Retriever (Aptum, Pick Cell Laboratories), and then rinsed three times for 5 min in milliQH_2_O. For tau (1:20,000; rabbit A0024, DAKO), IHC slides were immersed in 99% formic acid for 5 min, rinsed three times in milliQH_2_O, and then treated as per other slides. Slides were then incubated with an endogenous peroxidase blocking solution (50% methanol, 1% H_2_O_2_, diluted in mQH_2_O) for 20 min at RT. This was followed by three washes in PBS, and then slides were incubated with blocking buffer (10% normal goat serum in PBS) for 1 hour at RT. Primary antibodies mouse anti-ubiquitin (1:2000; MAB1510, Chemicon), CAB101 (1:500), and CAB102 (1:500) were applied for incubation overnight at 4°C.

To detect antibody binding, slides were washed in PBS with Triton X-100 for 5 min and then twice in PBS for 5 min each and incubated with biotinylated goat anti-mouse or anti-rabbit antibodies (Sigma-Aldrich) for 3 hours at RT. After further washing, they were incubated for 1 hour at RT with Sigma Extravidin peroxidase at 1:1000 dilution and then washed and incubated with the peroxidase substrate [3,3′-diaminobenzidine with 0.04% Ni(NH_4_)_2_(SO_4_)_2_] to develop the color change. Following PBS-milliQH_2_O washes (3 × 5 min each), the slides were dehydrated in a graded ethanol series followed by xylene and mounted under coverslips with DPX mounting medium.

Gingipain antibodies were optimized initially on formalin-fixed paraffin-embedded sections of gingival tissue collected from periodontal disease patients at the UCSF School of Dentistry under an IRB-approved protocol. Testing was then performed on MTG (from both postmortem control and AD human brains) and cerebellum (negative control). Specificity of antibody staining was demonstrated using positive and negative controls, secondary antibody only, isotype controls, and antigen pre-absorption. “No primary” antibody controls were negative for staining. Stained slides were scanned at 10× objective using a MetaSystems VSlide slide scanner, and brightfield images were analyzed for gingipain (and other markers) IR using the MetaMorph image analysis program. For each marker, the images were thresholded. Two thresholds for each core were determined: one to determine the total area of the core and the other to determine the total area of the thresholded region. To determine the load of staining per core, the thresholded area of staining was divided by the total core area. This analysis controlled for varying core sizes.

### IHC of human AD brain sections for neurons, astrocytes, and RgpB-IR

For 18E6 analysis, which recognizes a unique epitope within the immunoglobulin (Ig)–like domain of Arg-gingipain (RgpB) (*58*), IHC was performed using the Ventana Benchmark XT automated slide preparation system at the UCSF Brain Tumor Research Center tissue core. For immunoperoxidase staining, after tissue sections (5 μm thickness) were deparaffinized (at 75°C; EZ-Prep, Ventana Medical Systems), antigen retrieval was performed for 30 min [Cell Conditioning 1 (pH 8.5), Ventana Medical Systems] at 95° to 100°C. H_2_O_2_ (3%) (Thermo Fisher Scientific) was applied for 8 min to reduce background staining. Antibody 18E6 (University of Georgia Monoclonal Antibody Facility) was incubated at RT for 32 min at 1:10 dilution. Staining was developed using the UltraView Universal DAB Detection System (Ventana Medical Systems), and slides were counterstained with hematoxylin.

For immunofluorescence, sections were deparaffinized in xylene and rehydrated in a graded alcohol series. Heat-mediated antigen retrieval was performed with citric buffer (pH 6.0) (H-3300, Vector Laboratories). After PBS washes, sections were incubated in blocking solution, 5% donkey serum, and 0.3% Triton X-100 in PBS for 1 hour at RT. Sections were then incubated overnight at 4°C in primary antibodies anti-MAP2 (1:500; ab5392, Abcam), CAB101 (1:500), anti-IBA1 (1:1000; ab97120, Abcam), anti–β-amyloid, 17–24 (1:500; SIG-39200; 4G8, BioLegend), and anti-AT8 (1:2000; MN1020B, Thermo Fisher Scientific) in 3% donkey serum and 0.3% Triton X-100 in PBS. After PBS washes, slides were incubated with secondary antibody solution, either Alexa Fluor 647 goat anti-chicken (1:200; A21449, Life Technologies) and Alexa Fluor 488 donkey anti-rabbit (1:200; Jackson ImmunoResearch) mixed with anti–GFAP-Cy3 (1:250; MAB3402C3, Millipore) or Cy3-donkey anti-rabbit (1:200; Jackson ImmunoResearch), Alexa Fluor 488 donkey anti-mouse (1:200; Jackson ImmunoResearch), and Alexa Fluor 647 donkey anti-goat (1:200; Jackson ImmunoResearch) in PBS with 0.3% Triton X-100 for 2 hours at RT. Sections were washed in PBS and counterstained with 4′,6-diamidino-2-phenylindole (DAPI) (Invitrogen; D1306). Autofluorescence was quenched with TrueBlack Lipofuscin Autofluorescence Quencher 1:20 in 70% ethanol (catalog no. 23007, Biotium), and slides were mounted with ProLong Gold Antifade (P36930, Thermo Fisher Scientific). Coimmunofluorescence on *P. gingivalis* was performed by drying bacteria on SuperFrost Plus microscope slides. Bacteria were immersed in 4% paraformaldehyde for 10 min, washed three times in PBS, and incubated in formic acid for 7 min, followed by another three washes with PBS. Cells were exposed to anti–β-amyloid, 17–24 (1:500; SIG-39200; 4G8, BioLegend) and CAB101 (1:500) in 3% donkey serum and 0.3% Triton X-100 in PBS for 30 min at RT, followed by 30-min incubation in Cy3-donkey anti-rabbit (1:200; Jackson ImmunoResearch) and Alexa Fluor 488 donkey anti-mouse (1:200; Jackson ImmunoResearch) in PBS, counterstained with DAPI, and coverslipped with ProLong Gold Antifade (Thermo Fisher Scientific; P36930).

Histological analysis was performed on an Olympus BX61 motorized microscope. Fluorescence images were taken with a sCMOS camera (Zyla-5.5-USB3, Andor), and brightfield images were taken on a color charge-coupled device camera (DP27, Olympus). Images were processed for brightness and contrast correction, cropping, and addition of scale bars with CellSens 1.14 Dimension software (Olympus).

### Human brain Kgp IP and WB

Brain tissue from each subject sample (cortex, 100 mg) was homogenized on ice in 1 ml of B-PER lysis buffer (Thermo Fisher Scientific) with proteinase inhibitor cocktail (Millipore) and then kept on ice for 10 min. The bacteria control was prepared by pelleting 10^9^ bacteria by centrifugation at 5000*g* for 10 min. Then, the pellet was lysed in 1 ml of the same lysis buffer on ice for 10 min. All samples were then centrifuged at 16,000*g* for 20 min at 4°C, and the supernatant was collected. Protein concentration was measured using a Pierce BCA assay kit (Thermo Fisher Scientific). One milligram of total protein from each sample was denatured at 95°C for 5 min, and then an equal volume of antibody binding and washing buffer from the Dynabeads Protein G Immunoprecipitation Kit (Thermo Fisher Scientific) was added. For the bacteria control, 10^7^ bacteria were used. For the brain sample spiked with bacteria, 1 mg of total brain protein was mixed with bacterial lysate of 10^7^ bacteria. The samples were incubated with 10 μg of rabbit polyclonal CAB102 antibody with rotation overnight at 4°C. The next day, prewashed Dynabeads Protein G beads were incubated with 1 mg of bovine serum albumin (BSA) in binding buffer for 30 min and then washed three times with washing buffer. Then, samples were incubated with Dynabeads with rotation for 30 min at RT. Samples were washed four times with 200 μl of washing buffer using magnetic rack. Beads were then dissolved with 20 μl of elution buffer and 10 μl of NuPAGE LDS sample buffer and 50 mM dithiothreitol (DTT) and heated at 70°C for 10 min. Then, IP proteins were eluted from the magnetic beads using magnetic rack. Each sample (15 μl) was then subjected to SDS–polyacrylamide gel electrophoresis (PAGE) electrophoresis. SDS-PAGE gel was subjected to WB analysis by using Trans-blot Turbo transfer system (Bio-Rad) to transfer proteins to polyvinylidene difluoride (PVDF) membrane. The membrane was then rinsed with tris-buffered saline (TBS) and then blocked with blocking buffer from the Clean-Blot IP Detection Kit (Thermo Fisher Scientific) for 1 hour. The blot was then incubated with 1:1000 primary antibody CAB102 overnight with rocking in blocking buffer at 4°C. The blot was then washed three times with TBST buffer, 5 min each, and then incubated with 1:250 dilution of Clean-Blot detection reagent [horseradish peroxidase (HRP)] in blocking buffer for 1 hour at RT. Blot was then washed four times with TBST and then subjected to Pierce ECL detection reagent and ChemiDoc imaging system.

### qPCR analysis of *P. gingivalis* in human brain tissue

DNA was extracted and purified from postmortem cortex brain tissues following the protocol described in the Blood and Tissue Kit (Qiagen). Copy number of the *P. gingivalis* genome in brain DNA samples was determined by qPCR with *P. gingivalis*–specific *16S* primers [(ACCCTTTAAACCCAATAAATC (forward) and ACGAGTATTGCATTGAATG (reverse)] and fluorescent-labeled probe (CGCTCGCATCCTCCGTATTAC). The qPCR reaction mixture contained 100 ng of brain DNA, 0.5 μM primers/0.15 μM probe, and Kapa Fast qPCR Mix (Kapa Biosystems). PCR amplification was performed using the following cycling parameters: 3 min at 95°C, 50 cycles of 3 s at 95°C, and 30 s at 60°C. Copy number was determined from the standard curve generated using a synthetic template.

### Sequence analysis of *P. gingivalis* DNA amplified from human brain tissue

For sequencing, an approximately 1-kb region of the *P. gingivalis* genome encompassing the *hmuY* gene (except sequences corresponding to the first six amino acids) was amplified from 50 ng of brain DNA by PCR. PCR amplification (95°C/5 min; 95°C/20 s, 60°C/15 s, and 72°C/1 min for 35 cycles; 72°C/2 min) was performed using the KAPA HiFi HotStart ReadyMix PCR Kit (Kapa Biosystems) and primers [TTCTCCGCACTCTGTGCATT (forward) and AGCACTTCGATTCGCTCGAT(reverse)] designed to amplify the *hmuY* gene from the *P. gingivalis* genome. PCR products were run on 2% agarose gel, and DNA bands close to the expected size (based on the PCR product obtained from amplification of purified *P. gingivalis* DNA) were excised from the gel (Fig. 2F). DNA was extracted from the gel pieces following the protocol described in the Gel Extraction Kit (Qiagen). Approximately 5% of the eluted DNA was reamplified using the same *hmuY* PCR primers. Sequencing of reamplified PCR products was performed using nested primers (fig. S3).

### qPCR analysis of *P. gingivalis* in human CSF and saliva

CSF and matching saliva samples were obtained from PrecisionMed (Solana Beach, CA). Ten volunteer subjects, who were diagnosed with probable AD and met the criteria of having a Mini Mental Status Exam (MMSE) score of 20 or below, were serially enrolled in PrecisionMed’s IRB-approved protocol for CSF collection in November 2017 and donated CSF samples and matching saliva samples for *P. gingivalis* PCR analysis.

#### CSF PCR method

DNA was extracted from 50 μl of CSF using Puregene Core Kit A (Qiagen). The final DNA pellet was dissolved in 50 μl of DNA hydration buffer. A preamplification PCR assay (20 cycles) was performed with 5 μl of CSF DNA and *P. gingivalis hmuY* gene–specific H1.2 primers [GGTGAAGTCGTAAATGTTAC (H1.2 forward) and TTGACTGTAATACGGCCGAG (H1.2 reverse)]. A serial dilution of synthetic template DNA was also preamplified for the calculation of copy number. The preamplified PCR products were diluted, and a qPCR assay was performed with nested *hmuY* primers [GAACGATTTGAACTGGGACA (H1.1 forward) and AACGGTAGTAGCCTGATCCA (H1.1 reverse)] and a probe (/56-FAM/TTCTGTCTT/ZEN/GCCGGAGAATACGGC/3IABkFQ/). A serial dilution of the same synthetic template DNA was included in the qPCR assay to generate a standard curve and calculate starting copy number in CSF.

#### Saliva PCR method

DNA was extracted from 50 μl of saliva using Puregene Core Kit A (Qiagen). The final DNA pellet was dissolved in 50 μl of DNA hydration buffer. A qPCR assay was performed with 2 μl of saliva DNA and *hmuY* primers (H1.1) and the probe mentioned above. A serial dilution of synthetic template DNA was included in the qPCR assay to calculate starting copy number.

### qPCR analysis of *H. pylori* in human brain and CSF

The preamplification and qPCR protocol used for detection of *H. pylori* copy number in brain, CSF, and saliva samples were the same as those used for detecting the *P. gingivalis hmuY* gene copy number noted above. The qPCR primers and probe used for detection of *H. pylori* copy number have previously been described (*43*). We designed two primers [GATTAGTGCCCATATTATGGA (Hpy_outer_For) and CTCACCAGGAACTAATTTAC (Hpy_outer_Rev)] for the preamplification step. These primers amplified a 217-bp fragment encompassing the region amplified by the qPCR primers. A synthetic DNA of 240 bases encompassing the region amplified by outer primers was used as a control for the preamplification step.

### Effects of *P. gingivalis* infection on tau in SH-SY5Y cells

SH-SY5Y cells (~2.4 × 10^6^) were spin-inoculated with MOIs of 10, 50, and 100 with each of the following: *P. gingivalis* [American Type Culture Collection (ATCC) BAA-308] [wild type (WT)], *P. gingivalis KgP*Δ*Ig-B*, and *P. gingivalis* Δ*K/*Δ*RAB-A*. Uninfected SH-SY5Y cells were used as a control. SH-SY5Y cells and *P. gingivalis* strains were centrifuged at 1000*g* for 10 min at RT in Dulbecco’s modified Eagle’s medium (DMEM)/F12 supplemented with 2 mM l-glutamine and BSA (200 μg/ml), followed by incubation for 1, 4, and 8 hours, respectively, in a CO_2_ incubator. After the indicated incubation times, cells were collected and cell pellets were lysed with 250 μl of radioimmunoprecipitation assay buffer supplemented with protease inhibitor cocktail for 10 to 15 min. Total protein (16 μg) was used for WB. WBs were probed with tau monoclonal antibody TAU-5 (Thermo-MA5-12808). Glyceraldehyde-phosphate dehydrogenase (GAPDH) was used as an internal reference.

### Tau-441 incubation with gingipains and WB

Tau-441 (2N4R) (rPeptide, T-1001-1; molecular weight, 45.9 kDa) (2 μg) was reconstituted in 1% NH_4_OH and digested by 100, 10, 1, and 0.1 nM Kgp/RgpB in working buffer [20 nM sodium phosphate and 1 mM DTT (pH 7.5)]. Digestion reactions were performed for 1 hour at 37°C; reactions were stopped with protease inhibitor (P8340, Sigma-Aldrich). Proteins were separated by electrophoresis at 70 V for 15 min and then at 85 V for 1.5 hours on a 10% Criterion TGX precast gel (Bio-Rad, 5671033) (Bio-Rad, Criterion vertical electrophoresis cell) and electroblotted overnight onto a PVDF membrane at 10 V (Bio-Rad, Criterion Blotter). Blot was blocked with BLOTTO (87530, Thermo Fisher Scientific) for 1 hour and probed with primary antibody anti-Tau46 (13-6400, Thermo Fisher Scientific) at 1:1000 dilution in 3% BSA in TBS for 2 hours. Blot was then washed three times for 10 min each with TBST (28630, Thermo Fisher Scientific) and then incubated with secondary antibody HRP goat anti-mouse (1:50,000; 31439, Thermo Fisher Scientific) in 3% BSA in TBS for 30 min. After further washing, blot was washed three times for 10 min with TBST, and blot staining was visualized using chemiluminescence detection (34096; SuperSignal West Femto, Thermo Fisher Scientific).

### Liquid chromatography–MS/MS analysis of gingipain-generated tau cleavage products

Tau samples treated with 1 nM Kgp/RgpB or 10 nM Kgp/RgpB were analyzed by 1D Nano LC-MS/MS (JadeBio, San Diego, CA). A reversed-phase column (200 μm × 20 cm C_18_ 2.5 μm 130 Å) was generated in-house and coupled online to a Q Exactive mass spectrometer (Thermo Fisher Scientific, Bremen, Germany). Peptides were separated by a linear gradient from 95% buffer A [0.1% formic acid (FA) in water]/5% buffer B [0.1% FA in acetonitrile (ACN)] to 60% buffer A/40% buffer B over 150 min at 500 nl/min. The mass spectrometer was operated in a data-dependent TOP20 mode with the following settings: mass range, 400 to 2000 mass/charge ratio (*m/z*); resolution for MS^1^ scan, 70,000 full width at half maximum (FWHM); resolution for MS^2^ scan, 17,500 FWHM; isolation width, 3 *m/z*; NCE, 27; underfill ratio, 1%; dynamic exclusion, 15 s. Raw MS/MS spectra were searched against UniProt Human + *P. gingivalis* + decoy sequence databases. False discovery rate was <0.1% at the peptide level.

### Small-molecule gingipain inhibitor characterization

Structure-based design was used to develop a library of gingipain inhibitors, which were tested on purified Kgp and RgpB to assess potency and determine inhibition constants. The detailed chemical synthesis and structure of compounds in the relevant series of arginine gingipain inhibitors including COR286 can be found in International Patent Application PCT/US2015/054050 and PCT/US2016/061197. The detailed chemical synthesis and structure of compounds in the structural series for lysine gingipain inhibitors including COR119, COR271, and COR388 can be found in PCT/US2016/061197. The capacity of compounds to inhibit the activity of lysine or arginine gingipain was measured in a fluorogenic assay. The assay was performed in buffer [100 mM tris-HCl, 75 mM NaCl, 2.5 mM CaCl_2_, 10 mM cysteine, and 1% dimethyl sulfoxide (DMSO) (pH 7.5)] for 90 min at 37°C. Kgp, RgpB, and RgpA were isolated from culture of *P. gingivalis*, as described by Potempa and Nguyen (*39*). The fluorogenic substrate for Kgp was 10 μM Z-His-Glu-Lys-MCA and for RgpA and RgpB was 10 μM Boc-Phe-Ser-Arg-MCA. Trypsin buffer was 10 mM tris and 10 mM CaCl_2_ (pH 8.0), and the substrate was Z-Gly-Gly-Arg-AMC. COR286 has an IC_50_ of ≤20 pM on purified RgpB and an IC_50_ of 300 pM on structurally related RgpA but no significant inhibition of Kgp. COR119 has an IC_50_ of 10 nM on Kgp, with COR271 and COR388 having IC_50_ values of ≤50 pM on Kgp. All have no significant activity on RgpB. All compounds show negligible inhibition of trypsin with IC_50_ values of ≥10 μM. COR271 and COR388 were profiled more extensively on a series of cellular proteases, and no biologically meaningful activity (IC_50_ > 10 μM) was detected on these enzymes including cathepsin S, calpain, tryptase, thrombin, plasmin, FXa, FVIIa, BACE1, DPP4, proteasome, deubiquitinating peptidases, and caspase family enzymes.

A Morrison inhibition constant (*K*_i_) was determined for COR271 and COR388, and both display a *K*_i_ of <0.01 nM. Enzyme kinetic studies were performed to determine the mode of inhibition of the compounds by monitoring recovery of enzyme activity following dilution of existing enzyme/inhibitor complexes. COR119 is a reversible inhibitor, and COR271 and COR388 display irreversible binding kinetics. COR286 contains an identical catalytic binding site mechanism as COR271. The enzyme progress curves from this study were used to estimate *K*_off_ and *T*_1/2_ (half-life) of the reversible enzyme complex with COR119. Fitting the progress curve allows an estimate of *K*_off/obs_ (min^−1^) of 0.032 and *T*_1/2_ of 22 min for COR119. A *K*_off_ value for COR271 and COR388 cannot be calculated, as their binding is irreversible.

The COR553 activity probe was prepared by the copper-catalyzed azide-alkyne cycloaddition reaction (*106*) between an azide derivative of the irreversible Kgp inhibitor COR553 and an alkyne amide derivative of the Cy5 fluorophore. The COR553 probe forms an irreversible covalent bond with a catalytic cysteine residue in the active site of Kgp by displacement of a phenol leaving group.

### Effect of gingipain inhibitors on *P. gingivalis* toxicity in SH-SY5Y cells

Human neuroblastoma SH-SY5Y cells at 13 passages were cultured in complete medium [DMEM/F12 (Invitrogen) supplemented with 2 mM l-glutamine (Invitrogen), 10% heat-inactivated fetal bovine serum (10099141, Invitrogen), and 1% penicillin-streptomycin (Invitrogen)] in a 5% CO_2_ incubator (Thermo Fisher Scientific). Cells were seeded at a density of 2 × 10^4^ to 4 × 10^4^ cells per well (200 μl of 1 × 10^5^ to 2 × 10^5^ cells/ml) in 96-well black/flat-bottom plates (Greiner) manually coated with collagen type I and then incubated in a CO_2_ incubator at 37**°**C.

When cells reached 70 to 80% confluency (~6 × 10^4^ cells per well), they were challenged with *P. gingivalis* with or without COR271, COR286, moxifloxacin (32477, Fluka), doxycycline (D9891-5G, Sigma-Aldrich), or semagacestat (S1594, SELLECK) at various concentrations.

On the day of testing, the stock solution was diluted by eight serials of twofold dilution in DMSO (Sigma-Aldrich) in a sterile V-bottom 96-well plate (WIPP0280, Axygen) from well 2 to well 10. Well 11 contained DMSO only. From well 2 to well 11, the concentrations were 12.8, 6.4, 3.2, 1.6, 0.8, 0.4, 0.2, 0.1, 0.05, and 0 mg/ml. This was the compound mother plate, with each well containing 200× testing concentrations of compound in DMSO. Then, 6 μl from the mother plate was transferred into a 96-deep-well plate filled with 594 μl of complete medium–penicillin/streptomycin (1:100 dilution) to 2× testing concentration (128, 64, 32, 16, 8, 4, 2, 1, 0.5, and 0 μg/ml). This was the working solution.

*P. gingivalis* (ATCC BAA-308) was inoculated from −80°C stock onto a brain heart infusion agar (BD-211065). The plate was incubated for 72 hours at 37°C in an anaerobic workstation (YQX-II, Shanghai Yuejin). The atmosphere was kept at 80% N_2_, 10% CO_2_, and 10% H_2_.

On the day of testing, plates were processed in ambient atmosphere. Bacteria were harvested and suspended in complete medium–penicillin/streptomycin (without penicillin/streptomycin). Suspension was adjusted using a Siemens MicroScan turbidity meter (Siemens) to 0.5 turbidity, which is equivalent to ~6 × 10^8^ CFU/ml. Bacterial suspension was diluted in complete medium–penicillin/streptomycin to a final bacterial density of 6 × 10^8^ CFU/ml (for MOI of 1:1000) including one well with no bacteria as a negative control.

Cells in the testing plate were washed once with 200 μl of complete medium–penicillin/streptomycin. Then, 100 μl of working solution and 100 μl of bacterial suspension were added. The final testing concentrations were 64, 32, 16, 8, 4, 2, 1, 0.5, 0.025, and 0 μg/ml with 1% DMSO. The testing plates were incubated at 37°C in a 5% CO_2_ incubator for 24 hours.

Cell viability was determined using AlamarBlue (Invitrogen). Cells in the testing plates were washed twice using complete medium–penicillin/streptomycin to remove bacteria in the suspension. Then, 220 μl of AlamarBlue/medium mix (consisting of 200 μl of complete medium–penicillin/streptomycin and 20 μl of AlamarBlue) was added to each well of the testing plates. The testing plates were then incubated in a 37°C CO_2_ incubator for fluorescent reduced AlamarBlue to develop. The fluorescent signal from the reduced AlamarBlue (excitation, 530 nm/emission, 590 nm) was read after 6 hours, before saturation, on a SpectraMax M2e plate reader (Molecular Devices).

### Stereotactic injection of gingipains in mouse hippocampus

A 7-day study was designed to detect gingipain-induced hippocampal neurodegeneration with Fluoro-Jade C (FJC), a fluorescent stain that has been shown to exhibit maximum staining of degenerating neurons 1 week after a neurotoxic insult (*107*). Fifteen 8-week-old male BALB/c mice (Envigo) were used in the study. Animals were group-housed (*n* = 2 to 4 per cage) in plastic cages. Animals were maintained on a 12/12-hour light/dark cycle, with the RT maintained at 22° + 2°C and approximately 50% humidity, and received standard rodent chow and tap water ad libitum.

Mice were anesthetized using isoflurane (2%, 800 ml/min O_2_). Bupivacaine/epinephrine was used for local analgesia, and carprofen was used for perioperative/postoperative analgesia. A solution of RgpB (5 μg/ml) + Kgp (5 μg/ml) + 5 mM l-cysteine was prepared in sterile saline. Bilateral injections of 0.5 μl were made into coordinates from bregma: anteroposterior −2.0, lateral ±1.5, and ventral −1.4 mm from dura at a rate of 0.1 μl/min with a 5-min rest period using a Hamilton syringe (10-μl syringe with corresponding 30-gauge blunt tip needle; model no. 80308) and the stereotactic micromanipulator (Ultra Micro Pump III with Micro4 Controller, World Precision Instruments). When compound delivery was complete, the needle was left in place for 5 min and then withdrawn such that it took approximately 1 min to fully withdraw the needle.

Mice received a single administration of vehicle or drug 1.5 hours before stereotactic gingipain injection. Inhibitor-treated mice received COR271 (100 mg/kg) in PBS by oral gavage and COR286 (20 mg/kg) in 25% pluronic F127 subcutaneously at a dose volume of 5 and 10 ml/kg, respectively. Vehicle-treated mice received either PBS or pluronic.

Seven days later, mice were anesthetized with isoflurane (2%, 800 ml/min O_2_) and perfused with PBS. Brains were harvested, fixed in 10% formalin, embedded in paraffin, and sectioned at 5 μm.

Serial sections 200 μm apart through the entire hippocampus were stained with the FJC Ready-to-Dilute Staining Kit (Biosensis) according to the manufacturer’s protocol, and FJC-positive cells in the CA1 area were counted on an Olympus BX61 motorized microscope.

### Growth of *P. gingivalis* W83

*P. gingivalis* [W83 (ATCC, Rockville, MD), ΔKgp (Δ*kgp*), and ΔRgp (Δ*rgpArgpBΔ495-B Cm^r^*, *Em^r^*] (*57*, *58*) was streaked on tryptic soy broth (TSB) agar plates [5% sheep blood, supplemented with l-cysteine (0.5 mg/ml), hemin (5 μg/ml), and vitamin K (0.5 μg/ml)] and grown under anaerobic conditions at 37°C for 5 to 7 days. Samples were then inoculated in TSB with hemin, vitamin K, and l-cysteine (TSB-HKC) until mid-log phase OD_600_ (optical density at 600 nm) of 0.5 to 0.7. Bacteria were washed in PBS and prepared at a final concentration of 1 × 10^10^ cells/ml in PBS + 2% methylcellulose.

### *P. gingivalis* oral infection in mice

Experimental periodontitis was induced by ligature placement. During the procedure, mice were anesthetized with an intraperitoneal injection of ketamine (200 mg/kg; VetaKetam, Poland) and xylazine (10 mg/kg; Sedasin, Biowet, Poland), and the eyes were lubricated with ointment (Puralube Vet; PharmaDerm, Melville, NY). Next, a 5-0 silk ligature (Roboz Surgical Instrument Co., MD, USA) was tied around the upper maxillary left and right second molar. Suture was applied and tied gently to prevent damage to the periodontal tissue. The ligature was left in position for the entire experimental period so that inflammation could be constantly induced by colonization of bacteria inside of it.

#### Experiment 1

To study Aβ_1–42_ levels in the brains of orally infected mice, 40 (*n* = 8 per arm) 43- to 44- week-old female BALB/c mice were infected for 6 weeks every other day. For infection, 100 μl of the bacterial solution was applied topically to the buccal surface of the maxillae. COR119 in 2% DMSO/PBS was administered three times a day by subcutaneous injection starting on day 21 and continuing through day 42. Vehicle-treated animals received DMSO only. To further define the role of gingipains in the induction of brain Aβ_1–42_, mice were infected with *P. gingivalis* W83 (WT) or *P. gingivalis* lacking Kgp (Δ*kgp*) or the Rgp-null *P. gingivalis* mutant strain (ΔRgp) (*58*). After 6 weeks, the mice were euthanized and perfused with PBS, and brains were harvested and frozen in liquid nitrogen.

#### Experiment 2

To study the efficacy of gingipain inhibitors to decrease *P. gingivalis* infection of the brain, 100 (*n* = 10 per arm) 8-week-old female BALB/c mice were infected for 6 weeks every other day as described above. The mice received gingipain inhibitors or moxifloxacin for 5 weeks (days 36 to 70). COR271 was administered orally twice daily in PBS at 10 mg/kg; COR286 was administered subcutaneously twice daily in 25% pluronic F127 (10 mg/kg; Sigma-Aldrich, USA). Moxifloxacin (Sigma-Aldrich, USA) was administered subcutaneously twice daily in PBS at 10 mg/kg. Vehicle-treated animals received PBS or pluronic only. A group of mock-infected and *P. gingivalis* W83 (WT)–infected mice were euthanized on day 35 to gather baseline measurements before the start of treatment. After 10 weeks, *P. gingivalis* W83 (WT)–infected mice and mice infected with bacteria ± gingipain inhibitors or moxifloxacin were euthanized, and the brain and serum were harvested and frozen in liquid nitrogen_._

#### Experiment 3

To study the efficacy of gingipain inhibitors to decrease *P. gingivalis* infection of the brain, 70 (*n* = 10 per arm) 8-week-old female BALB/c mice were infected for 6 weeks every other day as described above. The mice received COR388 (3, 10, or 30 mg/kg) or COR271 (10 mg/kg) twice daily in PBS for 5 weeks (days 36 to 70) by oral administration. Vehicle-treated animals received PBS only. Mice were euthanized on day 35 or day 70, and one brain hemisphere and serum were frozen in liquid nitrogen, while one brain hemisphere was fixed in 10% formalin.

### Aβ ELISA

Brain samples (posterior half of the left hemisphere) were homogenized in radioimmunoprecipitation assay buffer (VWR), and Aβ_1–42_ was quantified with a Novex Mouse Beta Amyloid 1-42 (Aβ42) ELISA kit (Thermo Fisher Scientific, USA) according to the manufacturer’s specifications.

### ELISA assays for TNFα

Brain lysate was quantified for TNFα with ProcartaPlex Chemokine Convenience Panel 1 (Thermo Fisher Scientific) on a Luminex platform following the manufacturer’s protocol and with a V-PLEX Proinflammatory Panel 1 Mouse kit (Meso Scale Diagnostics, Rockville, MD). Results from both assays were corrected for protein content and normalized to the mock group, and the means of both assays were analyzed as a combined dataset.

### Endpoint PCR analysis of *P. gingivalis* in mice brain tissue

Bacterial DNA was extracted from mouse brains using DNeasy Blood & Tissue Kits (Qiagen, Germany) according to the manufacturer’s protocols. The concentration of DNA was measured using a NanoDrop 2000 (Thermo Fisher Scientific, USA). Bacterial DNA was amplified with 16*S* ribosomal RNA (rRNA) primers for the W83 strain of *P. gingivalis* [AGGCAGCTTGCCATACTGCG (forward) and ACTGTTAGCAACTACCGATGT (reverse)]. PCR amplification was conducted in a 12-μl reaction volume including 3 μl of brain DNA (80 ng of DNA), 6 μl of EconoTaq PLUS Green 2× Master Mix (Lucigen, USA), 0.6 μl (10 μM) of each primer (GenoMed, Poland), and 1.8 μl of H_2_O (Thermo Fisher Scientific, USA). Forty cycles of amplification were performed in a DNA thermal cycler (TProfessional TRIO, Biometra, Germany) consisting of 3 min for 95°C, 20 s for 95°C, 30 s for 57°C, 30 s for 72 °C, and 5 min for 72°C. The amplified product was identified by electrophoresis in a 1.5% agarose gel (BioShop, Canada). The DNA was stained with ethidium bromide, visualized under short wavelength transilluminator, and photographed in runVIEW imager (BIOCOMdirect, UK).

### Aβ_1–42_ binds to the surface of *P. gingivalis*

Recombinant Aβ (1-42) Ultra-Pure, Ammonium Hydroxide (rProtein) was prepared as stock solutions (1 mg/ml) in 1% NaNH_4_. *P. gingivalis* was washed in PBS and incubated at 10^8^ CFU/ml in Aβ (10, 30, and 100 μg/ml) for 1 hour at RT and ambient oxygen. For IHC, a 10-μl solution was dried on a SuperFrost Plus glass slide (VWR) fixed in 4% paraformaldehyde for 10 min. Slide was then rinsed with PBS and dH_2_O, exposed to formic acid, and, after washing with PBS, incubated for 2 hours in PBS, 0.3% Triton X-100, and primary antibodies CAB102 (1:1000) and 4G8 (1:1000; BioLegend). Fluorescence labeling was performed with Alexa Fluor 488 donkey anti-rabbit (1:200; Jackson ImmunoResearch) and Cy3 donkey anti-goat (1:200; Jackson ImmunoResearch) in PBS. Slides were mounted with ProLong Gold Antifade (Thermo Fisher Scientific). Images were taken on an Olympus BX61 microscope with a Zyla 5.5 sCMOS camera (Andor).

### Antimicrobial effects of Aβ on *P. gingivalis*

Recombinant Aβ (1-40, 1-42, 1-42 scrambled) Ultra-Pure, Ammonium Hydroxide (rProtein) was prepared as 0.2 mM stock solutions in 1% NaNH_4_. Aβ peptides were added to *P. gingivalis* cultures at a final concentration of 20 mM and kept at 37°C under anaerobic conditions for 24 hours. Cells were washed in PBS and stained with the LIVE/DEAD BacLight Bacterial Viability Kit (Thermo Fisher Scientific) according to the manufacturer’s protocol. Fluorescence intensity was quantified on a PerkinElmer Envision Plate reader.

### qPCR analysis of *P. gingivalis* in mouse brain tissue

DNA was extracted from brain tissue using the DNeasy Blood & Tissue Kit (Qiagen, Germany) according to the manufacturer’s protocol. TaqMan qPCR was performed with Kapa Probe fast qPCR Mix (Rox Low) on a Bio-Rad CFX96 Real-Time System C1000 Touch ThermalCycler with the forward (5′-AGCAACCAGCTACCGTTTAT-3′) and reverse (5′-GTACCTGTCGGTTTACCATCTT-3′) primers and 6-FAM-TACCATGTTTCGCAGAAGCCCTGA-TAMRA as the detection probe. The primers were based on single copy of *P. gingivalis* arginine–specific cysteine-proteinase gene (*108*). Duplicate samples were assayed in a total volume of 10 μl, containing 100 ng of template brain genomic DNA solution, TaqMan Universal PCR Master Mix (2×) (Kapa Biosystems, USA), and the specific set of primers (final concentration, 5 μM) and probe (final concentration, 4 μM) (GenoMed, Poland), corresponding to 562.5 nM of forward and reverse primers and 100 nM of the probe. After an initial incubation step of 2 min at 50°C and denaturation for 95°C for 20 s, 40 PCR cycles (95°C for 20 s and 60°C for 30 s) were performed. The number of copies of the *P. gingivalis* genome was calculated by matching *C*_q_ values with a standard curve prepared from serial dilutions of cultured *P. gingivalis* W83 (WT).

### Quantification of hippocampal Gad67^+^ interneurons

Anti-GAD67 antibody, clone 1G10.2 (MAB5406, MilliporeSigma), was used at a dilution of 1:2000 for IHC. Quantification of Gad67^+^ interneurons was performed with CellSens 1.5 Software. The area of the hilus was defined as the area between the blades of the dentate gyrus connected by a straight line on the open side. The number of cells on every 40th section through the hippocampus was counted. The results are presented as the number of cells per volume of tissue.

### Preparation of *P. gingivalis* lysates for gel electrophoresis

Bacteria (10^8^) were collected and centrifuged at 5000*g* for 10 min at 4°C. The supernatant was discarded. Bacterial cell pellet was lysed with 1 ml of B-PER lysis buffer (Thermo Fisher Scientific) on ice for 10 min and then centrifuged for 10 min at 14,000*g* at 4°C. The supernatant containing protein lysate was collected.

### COR553 activity probe labeling of Kgp

*P. gingivalis* lysate, purified Kgp, or human subgingival plaque samples were incubated with 1 μM of Cy5 probe COR553 for 1 hour at 37°C with shaking. For COR388-treated samples, 1 μM COR388 was added for 30 min at 37°C before the addition of COR553. Then, samples were denatured with NuPAGE LDS sample buffer (Thermo Fisher Scientific) containing 50 mM DTT at 95°C for 10 min and subjected to SDS-PAGE with Criterion 4 to 15% precast gel (Bio-Rad) and tris/glycine/SDS running buffer (Bio-Rad). Gel was run at 75 V for 10 min and then at 125 V for 1.5 hours, followed by Cy5 visualization with ChemiDoc imaging system (Bio-Rad).

### IP of Kgp labeled with COR553

For IP of Cy5-labeled Kgp, the samples were incubated with 10 μg of rabbit polyclonal CAB102 antibody with rotation overnight at 4°C, incubated with prewashed Dynabeads Protein G beads with rotation for 20 min at RT, washed, and magnetically separated. Beads were then dissolved with 20 μl of elution buffer and 10 μl of NuPAGE LDS sample buffer and 50 mM DTT and heated at 70°C for 10 min, eluting the IP proteins. Samples were then subjected to SDS-PAGE, and Cy5 probe signals were visualized with a ChemiDoc imaging system (Bio-Rad).

### WB analysis of COR553-labeled samples

After imaging with Cy5 detection, the same gels were transferred to PVDF membranes and immunoblotted with anti-Kgp antibody CAB102. Membranes were blocked with 3% BSA TBST buffer for at least 1 hour, incubated with 1:1000 CAB102 for 2 hours at RT or overnight at 4°C in blocking buffer, and visualized with goat anti-rabbit IgG HRP-conjugated antibody (#31462, Thermo Fisher Scientific) and chemiluminescent detection using SuperSignal West Femto (Thermo Fisher Scientific) and a ChemiDoc imaging system.

### Collection and processing of human saliva and subgingival plaque samples

Oral subgingival plaque and saliva samples were obtained from five human subjects with periodontal disease under an IRB-approved clinical protocol. An unstimulated saliva sample (about 1 ml) was obtained by collection into a sterile 15-ml falcon tube following a 2-min water rinse. Samples were collected at a consistent time of day to avoid diurnal effects and were kept cold during and following collection. After collection was complete, the cap of the tube was tightly screwed and transferred to −80°C.

Two subgingival plaque samples per site were collected from periodontal sites of four periodontal teeth with ≥6 mm pocket depth using Endodontic absorbent paper points (size, 40). The sampling sites were gently air-dried and isolated with cotton pellets to avoid saliva contamination. The paper points were inserted in the pockets for 30 s until resistance was felt. Paper points were held with pliers, removed from the site, and placed into prelabeled 1.5-ml microcentrifuge tubes. Samples were eluted from the paper points by placing them in 100 μl of B-PER lysis buffer in a low-bind 1.5-ml tube, flicking the tube at one flick per second for 30 s, discarding the paper point, and snap-freezing the samples in liquid nitrogen. Each plaque sample was processed in this manner separately but combined for analysis. Twenty microliters of the eluate was used for COR553 probe labeling, 5 μl was used for BCA protein determination, and 2 μl was used for qPCR.

### qPCR detection of *P. gingivalis* copy number in saliva and subgingival plaque of human subjects

DNA was extracted from 50 μl of Saliva using Puregene Core Kit A (Qiagen). The final DNA pellet was dissolved in 50 μl of DNA hydration buffer. A qPCR assay was performed with 2 μl of saliva DNA and *hmuY* primers [GAACGATTTGAACTGGGACA (H1.1 forward) and AACGGTAGTAGCCTGATCCA (H1.1 reverse)] and a probe (/56-FAM/TTCTGTCTT/ZEN/GCCGGAGAATACGGC/3IABkFQ/). A serial dilution of synthetic template DNA was included in the qPCR assay to calculate the copy number of *P. gingivalis* in saliva. qPCR was performed on 2 μl of neat eluate of subgingival plaque (no DNA extraction). The primers and methods were the same as those used for saliva above.

### Determination of Kgp-dependent growth of *P. gingivalis*

*P. gingivalis* [WT (ATCCBAA-308) and KgPΔIg-B] was inoculated from stocks into 20 ml of prereduced modified TSB medium [TSB + yeast extract (5 mg/ml), l-cysteine (0.5 mg/ml), hemin (5 μg/ml), and vitamin K1 (1 μg/ml)] and incubated at 37°C for 48 hours anaerobically in a Coy type C vinyl chamber. Prereduction of all solutions used was done by transferring the liquids to an anaerobic chamber for >16 hours immediately after autoclaving. On the day of the experiment, the primary culture was diluted to obtain OD of 0.2 to 0.25 using a Siemens MicroScan turbidity meter in the prereduced modified TSB medium and incubated for 6 hours to reach log phase (OD of approximately 0.5 to 0.6) at 37°C. Then, the bacteria were collected by centrifuging at 4000 rpm for 10 min and washed. Pellets were diluted to 3 × 10^8^ to 5 × 10^8^ CFU/ml, and 10 ml of these diluted cultures was transferred into conical tubes and centrifuged. The resultant pellet was resuspended using 10 ml of defined medium to assess growth. The defined medium consists of the following: salt base supplement [10.0 mM NaH_2_PO_4_, 10.0 mM KCI, 2 mM citric acid, 1.25 mM MgCl_2_, 20.0 μM CaCl_2_, 0.1 μM Na_2_MoO_4_, 25.0 μM ZnCl_2_, 50.0 μM MnCl_2_, 5.0 μM CuCl_2_, 10.0 μM CoCl_2_, and 5.0 μM H_3_BO_3_ (pH 7.0)] with 20 mM α-ketoglutarate, 3% BSA, hemin (5 μg/ml), and vitamin K (1 μg/ml). Fifty microliters of 100× COR388 stock prepared in DMSO was added to the bacterial suspension (3 × 10^8^ to 5 × 10^8^ CFU/ml) for each strain with a final concentration of 500 nM. Vehicle cultures were treated with 0.1% DMSO. The bacteria were incubated at 37°C in the anaerobic chamber, and OD was measured at 0, 21, and 43 hours to generate a time course of culture growth.

### Assessment of in vitro resistance of *P. gingivalis*

*P. gingivalis* (ATCC BAA-308) and *P. gingivalis* Kgp knockout were thawed, and a culture of OD_600_ = 1.2 (equals 3 × 10^9^ to 5 × 10^9^ CFU/ml) was prepared as described above. Resistance was assessed by incubation of 16 serial passages of *P. gingivalis* in the defined medium listed previously, and resistance to COR388 was performed simultaneously with cultures incubated with the broad-spectrum antibiotic moxifloxacin. Because COR388 does not completely inhibit *P. gingivalis* growth in vitro, we defined MIC as the minimum COR388 concentration that produced a partial inhibition cutoff, specifically >50% inhibition compared to nontreated cultures. Resistance was assessed in two standard methods, with the final data reported as an average of both methods. Cultures were first prepared with drug and moxifloxacin in a range of doses passaging each time for 17 passages and monitoring MIC with each passage. In a separate study, drug concentrations were gradually increased between passages. The lowest drug concentration that inhibited >50% growth was recorded as the MIC, inoculum from this passage used for the next passage, and assessed at a new drug concentration using the highest drug concentration that was sublethal and raising the concentrations throughout the test as needed.

### Statistical analysis

Data were analyzed with GraphPad Prism version 7.02 for Windows (GraphPad Software, La Jolla, CA, USA; www.graphpad.com). Outliers were detected with the ROUT method (*Q* = 0.2%) and removed from further analysis. Outliers were not removed from data presented in Fig. 1. To determine whether the data were normally distributed, we performed a Shapiro-Wilk test. If *P* values were below 0.05, then the data were considered nonparametric and analyzed by Mann-Whitney test or Kruskal-Wallis one-way analysis of variance (ANOVA) followed by Dunn’s post hoc test. Parametric data were analyzed by unpaired *t* test or by one-way ANOVA followed by Dunnett’s multiple comparisons test. Correlations were analyzed with Spearman’s correlation coefficient.

## Supplementary Material

http://advances.sciencemag.org/cgi/content/full/5/1/eaau3333/DC1

## SUPPLEMENTARY MATERIALS

Supplementary material for this article is available at http://advances.sciencemag.org/cgi/content/full/5/1/eaau3333/DC1 Fig. S1. CAB101 analysis of non-AD neurological disease brain microarrays. Fig. S2. RgpB IHC in hippocampal samples from nondemented and AD patients. Fig. S3. Sequencing of *P. gingivalis hmuY* PCR products from AD brains. Fig. S4. Sequencing of *P. gingivalis hmuY* PCR products from clinical AD CSF. Table S1. NVD003 AD and control TMA patient data. Table S2. NVD005 AD and control TMA patient data. Table S3. Tau fragments identified by MS after gingipain exposure. Table S4. Demographic information of patients with CP who donated saliva and subgingival plaque samples.
