# Supplementary material for: Porphyromonas gingivalis in Alzheimer’s disease brains: Evidence for disease causation and treatment with small-molecule inhibitors
Source: Sci Adv. 2019 Jan 23;5(1):eaau3333. doi: 10.1126/sciadv.aau3333 (PMC6357742; doi:10.1126/sciadv.aau3333)
Supplement: http://advances.sciencemag.org/cgi/content/full/5/1/eaau3333/DC1 [file supp_5_1_eaau3333__index.html]

Science Advances | Science Advances

## Supplementary Materials

**This PDF file includes:**

- Fig. S1. CAB101 analysis of non-AD neurological disease brain microarrays.
- Fig. S2. RgpB IHC in hippocampal samples from nondemented and AD patients.
- Fig. S3. Sequencing of *P. gingivalis hmuY* PCR products from AD brains.
- Fig. S4. Sequencing of *P. gingivalis hmuY* PCR products from clinical AD CSF.
- Table S1. NVD003 AD and control TMA patient data.
- Table S2. NVD005 AD and control TMA patient data.
- Table S3. Tau fragments identified by MS after gingipain exposure.
- Table S4. Demographic information of patients with CP who donated saliva and subgingival plaque samples.

Download PDF

**Files in this Data Supplement:**

- Adobe PDF - aau3333\_SM.pdf
