# Supplementary material for: Porphyromonas gingivalis in Alzheimer’s disease brains: Evidence for disease causation and treatment with small-molecule inhibitors
Source: Sci Adv. 2019 Jan 23;5(1):eaau3333. doi: 10.1126/sciadv.aau3333 (PMC6357742; doi:10.1126/sciadv.aau3333)
Supplement: http://advances.sciencemag.org/cgi/content/full/5/1/eaau3333/DC1 [file aau3333_SM.pdf]

Science Advances
AAAS

advances.sciencemag.org/cgi/content/full/5/1/eaau3333/DC1

Supplementary Materials for

Porphyromonas gingivalis in Alzheimer's disease brains: Evidence for disease causation and treatment with small-molecule inhibitors

Stephen S. Dominy*, Casey Lynch, Florian Ermini, Malgorzata Benedyk, Agata Marczyk, Andrei Konradi, Mai Nguyen, Ursula Haditsch, Debasish Raha, Christina Griffin, Leslie J. Holsinger, Shirin Arastu-Kapur, Samer Kaba, Alexander Lee, Mark I. Ryder, Barbara Potempa, Piotr Mydel, Annelie Hellvard, Karina Adamowicz, Hatice Hasturk, Glenn D. Walker, Eric C. Reynolds, Richard L. M. Faull, Maurice A. Curtis, Mike Dragunow, Jan Potempa

*Corresponding author. Email: sdominy@cortexyme.com

Published 23 January 2019, *Sci. Adv.* **5**, eaau3333 (2019)
DOI: 10.1126/sciadv.aau3333

This PDF file includes:

Fig. S1. CAB101 analysis of non-AD neurological disease brain microarrays.
Fig. S2. RgpB IHC in hippocampal samples from nondemented and AD patients.
Fig. S3. Sequencing of *P. gingivalis hmuY* PCR products from AD brains.
Fig. S4. Sequencing of *P. gingivalis hmuY* PCR products from clinical AD CSF.
Table S1. NVD003 AD and control TMA patient data.
Table S2. NVD005 AD and control TMA patient data.
Table S3. Tau fragments identified by MS after gingipain exposure.
Table S4. Demographic information of patients with CP who donated saliva and subgingival plaque samples.

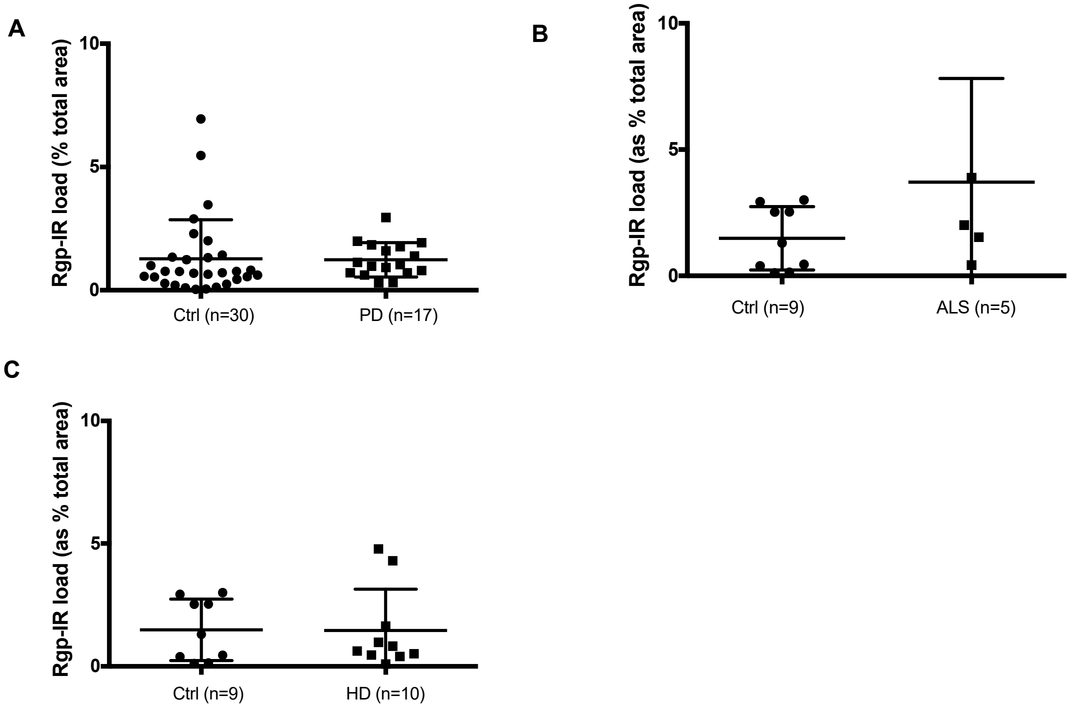

Fig. S1. CAB101 analysis of non-AD neurological disease brain microarrays. CAB101 staining of middle temporal gyrus for RgpB in (**A**) Parkinson's disease (PD), (**B**) amyotrophic lateral sclerosis (ALS), and (**C**) Huntington's disease (HD), reveals no significant difference when compared to control brain staining.

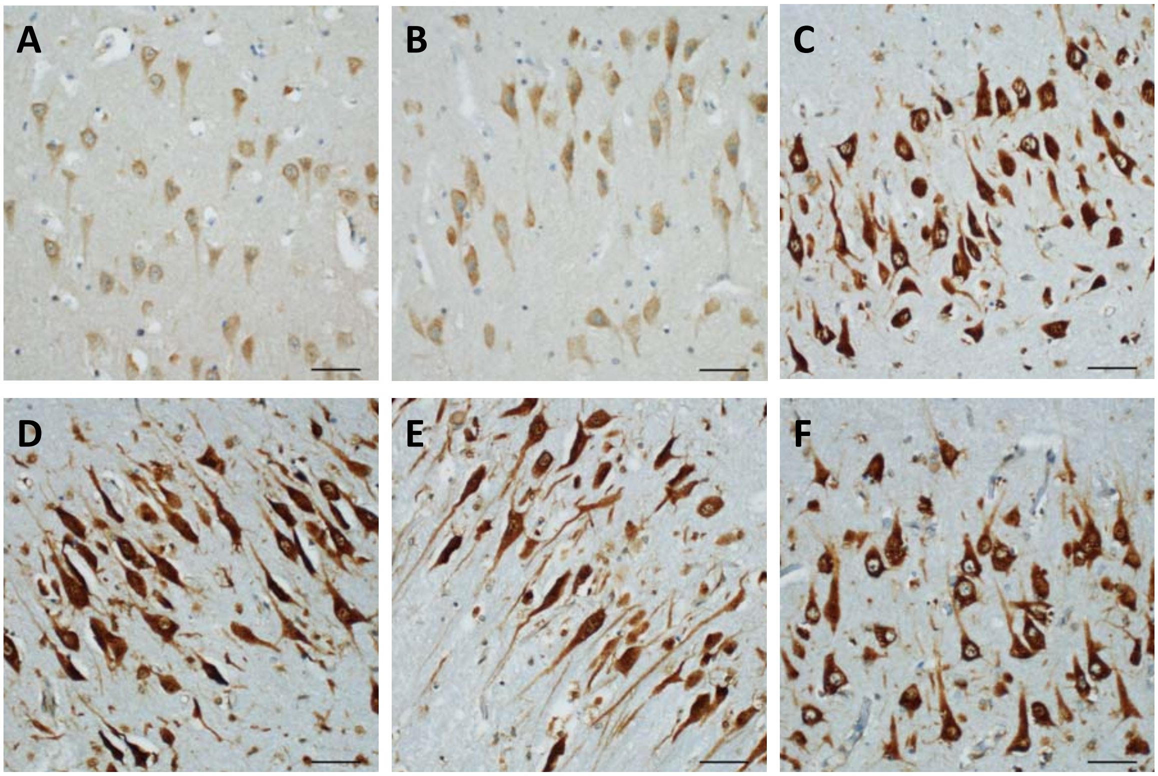

Fig. S2. RgpB IHC in hippocampal samples from nondemented and AD patients. RgpB IHC with monoclonal antibody 18E6 on pyramidal neurons of the CA3 region of the hippocampus was performed in a sample set obtained from a university brain bank with accompanying pathological diagnosis (n=14). Representative images of different levels of RgpB staining (brown color) in non-demented control (n=4) and AD diagnosed patients (n=10). Age, dementia status, and pathologic diagnosis for each subject are as follows: (**A**) 75-yo non-demented, no AD pathology. (**B**) 33-yo non-demented, very earliest forms of senile plaques. (**C**) 83-yo non-demented, minimal cognitive impairment (MCI), very mild AD pathology. (**D**) 66-yo demented, severe AD pathology. (**E**) 87-yo demented, severe AD pathology. (**F**) 61-yo demented, severe AD pathology. Scale bars 50 um.

A

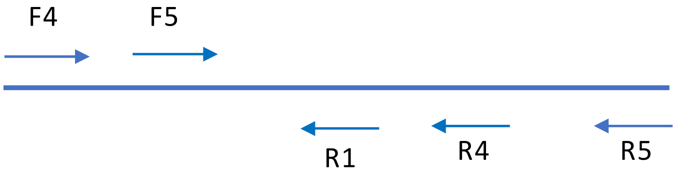

PCR and Sequencing primers

B

Disease State	Subject ID	PCR Primers	Sequencing Primers	% Identity
AD	83y	F5, R4	R1, R4	100, 100
AD	90y	F5, R4	R1, R4	99, 100
AD	80y	F5, R5	R5, R4	100,99

C

83y_R1
TTACCACTTTCGCCACAATTGAGACGAACGTCATAGCGGTGAAGAGCCATGTCCCAGTTCAAATCGTTCTTA
TAGTCGGTAACATTTACGACTTCACCTTTGGAAAAAGAGAAATACTGCCACGTTTCGTATTTCGAAGCATCG
ATAGTTACGGTTTTGGTTACTGT

83y_R4
AGCAGGCCGATGAGGGCCTGCTTTGTTACTACACTTTTCATATTTCCCTCTTAACTTATTTAACGGGGTATG
TATAAGTGAAAGTGATGACTCCTTTTTTGAGTTCTGCATCCTGATAGTCAGTGAACTGCACTTTGGCAATAT
TACCATCAGCACCACGAACGAAGAAGACTCTTTTGCTCAGCTTGTAAGTGGGACCGGCAGGACCGTGAGAGA
ATTCCAGCCAACCACCTGAAGCAAATCCCTGTGCGTTCTTCTTGCCGGTAATCACTTCGCTGAAGCCCTGTT
CTTCATATTCCATCTGATGACCATCAGGTCCCATTTCGTACTTGACTGTAATACGGCCGAGAACATCTACAG
TATATCCGTCTGTCGGAACGGTAGTAGCCTGATCCATTTCTGTCTTGCCGGAGAATACGGCACCACCTTTTC
CCTTACCACTTTCGCCACAATTGAGACGAACGTCATAGCGGTGAAGAGCCATGTCCCAGTTCAAATCGTTCT
TATAGTCGGTAACATTTACGACTTCACCTTTGGAAAAAGAGAAATACTGCCACGTTTCGTATTTCGAAGCAT
CGATAGTTACGGTTTTGGTTACTG

90y_R4
TACTTATACTAAAGAAAAGCAGGCCGATGAGGGCCTGCTTTGTTACTACACTTTTCATATTTCCCTCTTAAC
TTATTTAACGGGGTATGTATAAGTGAAAGTGATGACTCCTTTTTTGAGTTCTGCATCCTGATAGTCAGTGA
ACTGCACTTTGGCAATATTACCATCAGCACCACGAACGAAGAAGACTCTTTTGCTCAGCTTGTAAGTGGGAC
CGGCAGGACCGTGAGAGAATTCCAGCCAACCACCTGAAGCAAATCCCTGTGCGTTCTTCTTGCCGGTAATCA
CTTCGCTGAAGCCCTGTTCTTCATATTCCATCTGATGACCATCAGGTCCCATTTCGTACTTGACTGTAATAC
GGCCGAGAACATCTACAGTATATCCGTCTGTCGGAACGGTAGTAGCCTGATCCA8100TTTCTGTCTTGCCG
GAGAATACGGCACCACCTTTTCCCTTACCACTTTCGCCACAATTGAGACGAACGTCATAGCGGTGAAGAGCC
ATGTCCCAGTTCAAATCGTTCTTATAGTCGGTAACATTTACGACTTCACCTTTGGAAAAAGAGAAATACTGC
CACGTTTCGTATTTCGAAGCATCGATAGTTACGGTTTTGGTTACTGCTT

90y_R1
CCTTTTCCCTTACCACTTTCGCCACAATTGAGACGAACGTCATAGCGGTGAAGAGCCATGTCCCAGTTCAAA
TCGTTCTTATAGTCGGTAACATTTACGACTTCACCTTTGGAAAAAGAGAAATACTGCCACGTTTCGTATTTC
GAAGCATCGATAGTTACGGTTTTGGTTACTGCTTCGGGTGTGGAA

80y_R5
ATACTTATACTAAAGAAAAGCAGGCCGATGAGGGCCTGCTTTGTTACTACACTTTTCATATTTCCCTCTTAA
CTTATTTAACGGGGTATGTATAAGTGAAAGTGATGACTCCTTTTTTGAGTTCTGCATCCTGATAGTCAGTG
AACTGCACTTTGGCAATATTACCATCAGCACCACGAACGAAGAAGACTCTTTTGCTCAGCTTGTAAGTGGGA
CCGGCAGGACCGTGAGAGAATTCCAGCCAACCACCTGAAGCAAATCCCTGTGCGTTCTTCTTGCCGGTAATC
ACTTCGCTGAAGCCCTGTTCTTCATATTCCATCTGATGACCATCAGGTCCCATTTCGTACTTGACTGTAATA
CGGCCGAGAACATCTACAGTATATCCGTCTGTCGGAACGGTAGTAGCCTGATCCATTTCTGTCTTGCCGGAG
AATACGGCACCACCTTTTCCCTTACCACTTTCGCCACAATTGAGACGAACGTCATAGCGGTGAAGAGCCATG
TCCCAGTTCAAATCGTTCTTATAGTCGGTAACATTTACGACTTCACCTTTGGAAAAAGAGAAATACTGCCAC
GTTTCGTATTTCGAAGCATCGATAGTTACGGTTTTGGTTACTG

80y_R4
TACTTATACTAAAGAAAAGCAGGCCGATGAGGGCCTGCTTTGTTACTACACTTTTCATATTTCCCTCTTAAC
TTATTTAACGGGGTATGTATAAGTGAAAGTGATGACTCCTTTTTTGAGTTCTGCATCCTGATAGTCAGTGA
ACTGCACTTTGGCAATATTACCATCAGCACCACGAACGAAGAAGACTCTTTTGCTCAGCTTGTAAGTGGGAC
CGGCAGGACCGTGAGAGAATTCCAGCCAACCACCTGAAGCAAATCCCTGTGCGTTCTTCTTGCCGGTAATCA
CTTCGCTGAAGCCCTGTTCTTCATATTCCATCTGATGACCATCAGGTCCCATTTCGTACTTGACTGTAATAC
GGCCGAGAACATCTACAGTATATCCGTCTGTCGGAACGGTAGTAGCCTGATCCATTTCTGTCTTGCCGGAGA
ATACGGCACCACCTTTTCCCTTACCACTTTCGCCACAATTGAGACGAACGTCATAGCGGTGAAGAGCCATGT
CCCAGTTCAAATCGTTCTTATAGTCGGTAACATTTACGACTTCACCTTTGGAAAAAGAGAAATACTGCCACG
TTTCGTATTTCGAAGCATCGATAGTTACGGTTTTGGTTACTGCT

D

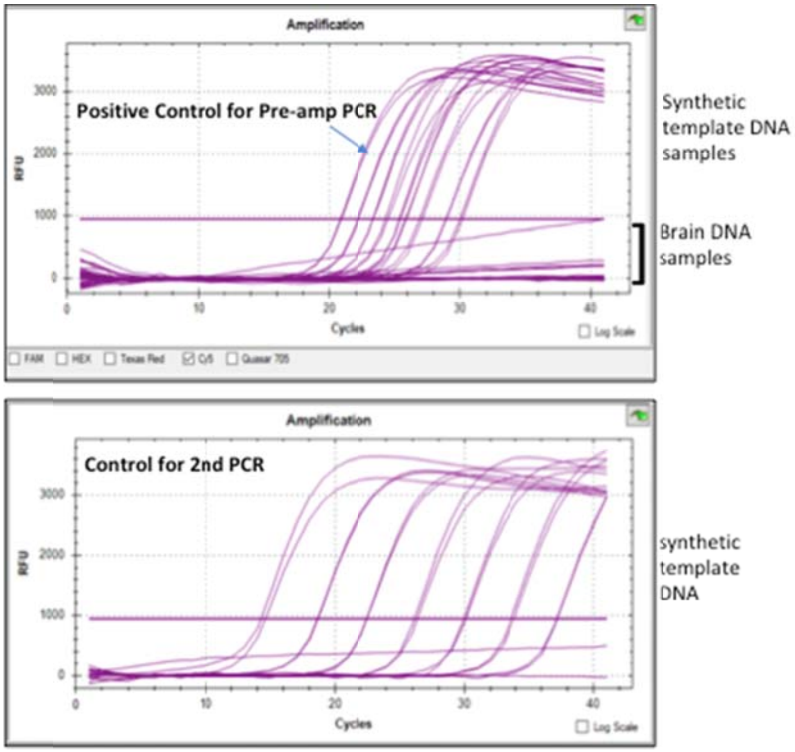

Fig. S3. Sequencing of *P. gingivalis hmuY* PCR products from AD brains. (**A**) PCR-sequencing strategy for the *P. gingivalis hmuY* gene. (**B**) Data table includes subject disease state and subject ID used in Fig. 3 of main text, sequencing primers used, and sequencing identity of PCR products to *P. gingivalis hmuY* DNA sequence. (**C**) PCR product sequences for each AD subject. (**D**) *H. pylori* synthetic template DNA samples used as positive control for PCR of human AD brains.

A

Subject ID	MMSE Score	Age	PCR primers	Sequencing Primers	% Identity
AD2	15	59	H1.2 and H1.1	H1.1 For and Rev	100, 99
AD4	17	72	H1.2 and H1.1	H1.1 For and Rev	100, 99
AD6	18	60	H1.2 and H1.1	H1.1 For and Rev	100, 97
AD7	18	60	H1.2 and H1.1	H1.1 For and Rev	100, 99
AD8	18	59	H1.2 and H1.1	H1.1 For and Rev	100, 100
AD9	18	62	H1.2 and H1.1	H1.1 For and Rev	99, 99
AD10	20	61	H1.2 and H1.1	H1.1 For and Rev	99, 98

B

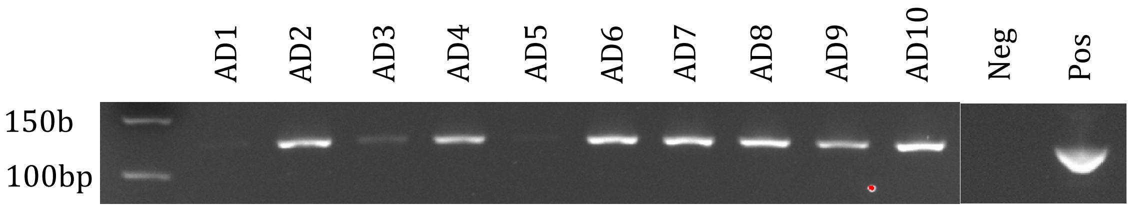

C

AD2_H1.1 For
TGGTAAGGGAAAAGGTGGTGCCGTATTCTCCGGCAAGACAGAAATGGATCAGGCTACTA
CC
AD2_H1.1 Rev
CCTTTTCCTTACCACTTTCGCCACAATTGAGACGAACGTCATAGCGGTGAAGAGCCATGT
CCCAGTTCAAATCGTTA
AD4_H1.1 For
TGGTAAGGGAAAAGGTGGTGCCGTATTCTCCGGCAAGACAGAAATGGATCAGGCTACTA
CCGTTAA
AD4_H1.1 Rev
CCTTTTCCTTACCACTTTCGCCACAATTGAGACGAACGTCATAGCGGTGAAGAGCCATGT
CCCAGTTCAAATC

AD6_H1.1 For
TGGTAAGGGAAAAGGTGGTGCCGTATTCTCCGGCAAGACAGAAATGGATCAGGCTACTACC

AD6_H1.1 Rev
CCTTTTCCCTTACCACTTTNNGCCACAATTGAGACGAACGTCATAGCGGTGAAGAGCCATGTCCCAGTTCAAATCGTTAAAA

AD7_H1.1 For
GGTAAGGGAAAAGGTGGTGCCGTATTCTCCGGCAAGACAGAAATGGATCAGGCTACTACC

AD7_H1.1 Rev
CGGCACCACCTTTTCCTTACCACTTTCGCCACAATTGAGACGAACGTCATAGCGGTGAAGAGCCATGTCCCAGTTCAAATC

AD8_H1.1 For
TGGTAAGGGAAAAGGTGGTGCCGTATTCTCCGGCAAGACAGAAATGGATCAGGCTACTACCG

AD8_H1.1 Rev
CCTTTTCCTTACCACTTTCGCCACAATTGAGACGAACGTCATAGCGGTGAAGAGCCATGTCCCAGTTCAAATC

AD9_H1.1 For
TTCGTCTCATTGTGGCGAAAGTGGTAAGGGAAAAGGTGGTGCCGTATTCTCCGGCAAGACAGAAATGGATCAGGCTACTACCGT

AD9_H1.1 Rev
CCTTTTCCTTACCACTTTCGCCACAATTGAGACGAACGTCATAGCGGTGAAGAGCCATGTCCCAGTTCAAATCGTTCC

AD10_H1.1 For
GTTCGTCTCATTGTGGCGAAAGTGGTAAGGGAAAAGGTGGTGCCGTATTCTCCGGCAAGACAGAAATGGATCAGGCTACTACCG

AD10_H1.1 Rev
CGGCACCNCCTTTTCCTTACCACTTTCGCCACAATTGAGACGAACGTCATAGCGGTGAAGAGCCATGTCCCAGTTCAAATCGTTCC

Fig. S4. Sequencing of *P. gingivalis hmuY* PCR products from clinical AD CSF. (**A**) Data table includes subject ID used in Fig. 4 of main text, Mini Mental Status Exam (MMSE) score, age and sequence identity of PCR products to *P. gingivalis hmuY* DNA sequence. (**B**) PCR products detecting *P. gingivalis* from CSF run on agarose gel including a negative-control and a positive-control (a synthetic DNA template). (**C**) Sequences of PCR product for each AD subject.

Table S1. NVD003 AD and control TMA patient data.

Case No.	Diagnosis	Age	M/F	PMD (hr)	Pathology - Diagnosis
**AZ34**	AD	74	F	18	CERAD: Definite Alzheimer's disease Atrophy: mod-2, Tangles: mod-2, Plaques: mod-2, ARP: C
**AZ37**	AD	83	M	4	CERAD: Probable Alzheimer's disease Atrophy: severe-3, Tangles: mod-2, Plaques: mild-1,
**AZ38**	AD	80	M	5.5	CERAD: Definite Alzheimer's disease Braak: V; Atrophy: mod-2, Tangles: mod-2, Plaques: frequent-3,
**AZ39**	AD	74	M	12	CERAD: Definite Alzheimer's disease Braak: VI Atrophy: mild-1, Tangles: mod-2, Plaques: mod-2,
**AZ42**	AD	60	M	7	CERAD: Definite Alzheimer's disease Braak: VI Atrophy: severe-3, Tangles: mild-1, Plaques: freq-3,
**AZ43**	AD	80	M	21	CERAD: Probable Alzheimer's disease Atrophy: mild-1, Tangles: mod-2, Plaques: mod-2,
**AZ45**	AD	82	M	4.5	CERAD: Probable Alzheimer's disease Atrophy: mild-1, Tangles: mod-2, Plaques: mod-2,
**AZ46**	AD	82	F	22	CERAD: Probable Alzheimer's disease Atrophy: mild-1, Tangles: mod-2, Plaques: mod-2,
**AZ52**	AD	68	F	36	CERAD: Definite Alzheimer's disease Atrophy: severe-3, Tangles: freq-3, Plaques: freq-3,
**AZ55**	AD	51	M	4	CERAD: Probable Alzheimer's disease Atrophy: mild-1, Tangles: mod-2, Plaques: mild-1,
**AZ57**	AD	82	F	14.5	CERAD: Possible Alzheimer's disease Atrophy: mild-1, Tangles: mod-2, Plaques: scant-1,
**AZ58**	AD	75	M	20	CERAD: Definite Alzheimer's disease Atrophy: severe-3, Tangles: freq-3 Plaques: freq-3,
**AZ59**	AD	83	M	15	CERAD: Possible Alzheimer's disease Atrophy: mild, Tangles: scant-1, Plaques: scant-1,
**AZ61**	AD	87	F	7.5	Definite Alzheimer's disease
**AZ64**	AD	67	M	8	CERAD: Definite Alzheimer's disease Atrophy: mild-1, Tangles: mod-2, Plaques: freq-3, ARP: C
**AZ65**	AD	77	F	16	CERAD: Alzheimer's disease (definite) Atrophy: severe, Tangles: mod, Plaques: numerous,
**AZ68**	AD	68	F	7	CERAD: Alzheimer's disease. Atrophy:severe, Tangles:numerous, Plaques:numerous,
**AZ71**	AD	62	F	6	CERAD: Definite Alzheimer's disease Braak: VI; Atrophy: 2/3, Tangles: 3/3, Plaques: 3/3,
**AZ72**	AD	70	F	7	CERAD: Indicative of Alzheimer's disease Braak: V; Atrophy: 0/3, Tangles: 1/3, Plaques: 3/3,
****AZ73**	AD	87	F	14.5	Mixed Pathology: Alzheimer's disease & Cortical Lewy Body Dis. Braak: IV, Atrophy: 1/3, Tangles: 2/3, Plaques: 2/3,
**AZ74**	AD	85	F	16	CERAD: definite Alzheimer's disease. Braak: VI, Atrophy: 2/3, Tangles: 2/3, Plaques: 3/3,
**AZ75**	AD	81	M	25	CERAD: definite Alzheimer's disease. Braak: 5/6, Atrophy: 1/3, Tangles: 2/3, Plaques: 3/3,
**AZ77**	AD	81	F	16	CERAD: probable Alzheimer's disease. Braak: 4/6, Atrophy: 3/3, Tangles: 3/3, Plaques: 2/3,
**AZ80**	AD	77	M	4.5	CERAD: Definite Alzheimer's disease Braak: 6/6; Atrophy: 3/3, Tangles: 3/3, Plaques: 3/3,
**AZ81**	AD	82	F	18	CERAD: definite Alzheimer's disease. Braak: 6/6, Atrophy: 3/3, Tangles: 3/3, Plaques: 3/3,
**AZ82**	AD	80	F	18	CERAD: diagnostic of Alzheimer's disease. Braak: 6/6, Atrophy: 3/3, Tangles: 3/3, Plaques: 3/3,
**AZ83**	AD	60	F	16	CERAD: Alzheimer's disease. Braak: 6/6, Atrophy: 3/3, Tangles: 3/3, Plaques: 3/3,
**AZ87**	AD	73	M	5	CERAD: Definite Alzheimer's disease Braak: 5/6; Atrophy: 2/3, Tangles: 3/3, Plaques: 3/3,
**AZ89**	AD	80	F	25	CERAD: Definite Alzheimer's disease Braak: 6/6; Atrophy: 3/3, Tangles: 3/3, Plaques: 3/3, ARP: C
**H190**	Control	72	F	19	
**4734**	Control	79	M	8	
**H123**	Control	78	M	7.5	
**H136**	Control	75	M	13	
**H155**	Control	61	M	7	
**H152**	Control	79	M	18	
**4680**	Control	80	M	12	
**H160**	Control	77	M	23	
**H198**	Control	67	F	27	
**H145**	Control	54	M	8	
**H196**	Control	85	M	15	
**H144**	Control	76	M	18.5	
**H202**	Control	83	M	14	
**H156**	Control	89	M	19	
**H148**	Control	64	M	7	
**H164**	Control	73	M	13	
**6013**	Control	69	F	11.5	
**H121**	Control	64	F	5	

**H122**	Control	72	F	9
**02F/393**	Control	87	F	11
**H187**	Control	98	F	15
**H180**	Control	73	M	33
**H150**	Control	78	M	11
**H153**	Control	76	M	8
**H188**	Control	83	M	17
**H191**	Control	77	M	20
**H127**	Control	59	F	21
**H139**	Control	73	M	5.5
**H137**	Control	77	M	12

Table S2. NVD005 AD and control TMA patient data.

Case No.	Diagnosis	Age	M/F	PMD (hr)	Pathology - Diagnosis
**A10**	AD	64	M		Dementia of the Alzheimer's type
**A12**	AD	72	M	9	Confident diagnosis of Alzheimer's disease
**A13**	PD	79	M	36	Developing idiopathic Parkinson's disease
**AZ14**	AD	77	F	15	Appearances are those of a dementia of Alzheimer's type
**AZ15**	AD/PD	80	M	3.5	Cerebral cortical findings> AD; SN > idiopathic PD
**AZ24**	AD	82	F	97	CERAD: Definite Alzheimer's disease Atrophy: mild-1, Tangles: mod-2, Plaques: freq -3, ARP: C
**AZ33**	AD	65	M	20	CERAD: Definite Alzheimer's disease Atrophy: mid-1, Tangles: mild-1, Plaques: mod-2, ARP: C
**AZ60**	AD	83	M	8	Alzheimer's disease
**AZ62**	AD/Epil	80	F	11	Alzheimer's disease [+ history of epilepsy]
**AZ78**	AD	87	F	7	CERAD: probable Alzheimer's disease. Braak: 3/6, Atrophy: 2/3, Tangles: 2/3, Plaques: 1/3, ARP: B
**AZ85**	AD	85	M	57	CERAD: probable Alzheimer's disease Braak: 4/6, Atrophy: 0/3, Tangles: 2/3, Plaques: 1/3, ARP: B
**AZ86**	AD	92	M	8.5	CERAD: possible Alzheimer's disease Braak: 3/6, Atrophy: 0/3, Tangles: 1/3, Plaques: 1/3, ARP: A
**AZ88**	AD	83	M	21	CERAD: Definite Alzheimer's disease Braak: 4/6; Atrophy: 2/3, Tangles: 3/3, Plaques: 3/3, ARP: C
**AZ89**	AD	80	F	25	CERAD: Definite Alzheimer's disease Braak: 6/6; Atrophy: 3/3, Tangles: 3/3, Plaques: 3/3, ARP: C
**AZ90**	AD	73	M	4	CERAD: Definite Alzheimer's disease Braak: 4/6; Atrophy: 3/3, Tangles: 3/3, Plaques: 3/3, ARP: C
**AZ91**	AD	80	M	29	CERAD: Definite Alzheimer's disease Braak: 5/6; Atrophy: 2/3, Tangles: 3/3, Plaques: 3/3, ARP: C
**AZ92**	AD	93	F	11.5	CERAD: Probable Alzheimer's disease Braak: 4/6, Atrophy: 3/3, Tangles: 3/3, Plaques: 3/3, ARP: B
**AZ93**	AD	83	M	15	CERAD: Probable Alzeimer Disease (mod paque density); Braak Stage V
**AZ95**	AD	69	M	12	CERAD: Alzheimer's disease. Braak: 5/6, Atrophy: 3/3, Tangles: 2/3, Plaques: 3/3, ARP: C
**AZ96**	AD	74	F	8.5	CERAD: Alzheimer's disease Braak: 5/6; Atrophy: 3/3, Tangles: 3/3, Plaques: 3/3, ARP: C
**AZ98**	AD	91	F	20.5	Alzheimer's disease
**AZ99**	AD	94	F	8.5	Alz-type neuropath. change (A3, B3, C2), Braak V-VI; small vessel cerebrovascualr wth focal lacunar infarction; focal cerebral amyloid angiopathy
**AZ101**	AD	75	M	12.5	AD neuopath. change (A3, B3, C2), Braak VI; cerebral amyloid angiopathy; old infarction in R temporo-occipital; small vessel cerebrovascular disease
**AZ102**	AD	84	F	14.5	Alz-type neuopathological change (A3, B2, C2), Braak IV; hyaline arteriosclerosis
**AZ103**	AD	87	M	<24	Alz-type neuropathiclogic change (A3, B2, C2), Braak IV; cerebral amyloid angiopathy; small vessel disease
**AZ107**	AD	86	M		Intermediate AD change (A2, B3, C1), Braak VI; Amygdala predominant Lewy body diseease; deep small vessel disease; cerebral amyloid angiopathy
**AZ108**	AD	94	F	11.5	Consistent with Alzheimer’s disease (NIA-AA score A3 B3 C2, high degree of AD change), LBD, amygdala predominat, cerebral amyloid angiophaty, hyaline arteriolosclerosis
**AZ109**	AD	90	F	31	Consistent with Alzheimer’s disease (NIA-AA score A3 B2 C1, intermediate AD change), cerebral amyloid angiophaty, hyaline arteriolosclerosis
**AZ110**	AD	86	F	15	Consistent with Alzheimer’s disease (NIA-AA score A3 B3 C2, high AD change); hippocampal sclerosis with associated TDP-43 path; LBD, difuse; focal cerebral amyloid angiopathy; small
**H151**	control	64	F	5	
**H159**	control	53	M	16.5	

**H165**	control	43	F	26
**H168**	control	63	M	9
**H169**	control	81	M	24
**H170**	control	60	M	17
**H174**	control	59	M	24.5
**H177**	control	22	M	21
**H183**	control	61	M	10
**H184**	control	35	M	20
**H185**	control	50	M	28-31
**H186**	control	68	M	21
**H192**	control	65	F	23-28
**H194**	control	68	M	22.5
**H195**	control	65	M	18
**H197**	control	19	F	40
**H200**	control	56	M	23
**H209**	control	48	M	23
**H211**	control	41	M	8
**H215**	control	67	F	23.5
**H226**	control	73	F	48
**H230**	control	57	F	32
**H231**	control	65	M	8
**H238**	control	63	F	16
**H239**	control	64	M	15.5
**H240**	control	73	M	26.5
**H242**	control	61	M	19.5
**H243**	control	77	F	13
**H245**	control	63	M	20

Table S3. Tau fragments identified by MS after gingipain exposure.

No. observations following 1 nm Kgp/Rgp	No. observations following 10 nm Kgp/Rgp	Peptide sequence	Score	Uniprot protein accession numbers*
2	19	HLSNVSSTGSIDMVDSPQLATLADEVSASLAK	32.14	A0A0G2JMX7,A0A024R9Y0,B4DSE3,B4DSB3,A0A024RA17,A0A0G2JPD5,A0A0G2JS76,A0A024R9Y1, P10636,A0A024RA19,I3L170,A0A0G2JQJ7,B3KTM0
	9	qAAAQPHTEIPEGTTAEEAGIGDTPSLEDEAAGHVTQAR	31.34	A0A024RA17
1	15	QAAAQPHTEIPEGTTAEEAGIGDTPSLEDEAAGHVTQAR	29.75	A0A024RA17
	20	KDQGGYTMHQDQEGDTDAGLKESPLQTPTEDGSEEPGSETSDAK	28.61	A0A0G2JMX7,A0A024RA19,A0A0G2JQJ7,A0A024RA17,A0A0G2JPD5,A0A0G2JS76,A0A024R9Y1, P10636
3	3	HLSNVSSTGSIDMVDSPQLATLADEVSASLAKQGL	28.58	A0A0G2JMX7,A0A024R9Y0,B4DSE3,B4DSB3,A0A024RA17,A0A0G2JPD5,A0A0G2JS76,A0A024R9Y1, P10636,A0A024RA19,I3L170,A0A0G2JQJ7,B3KTM0
1	7	HLSNVSSTGSIDmVDSPQLATLADEVSASLAK	28.58	A0A0G2JMX7,A0A024R9Y0,B4DSE3,B4DSB3,A0A024RA17,A0A0G2JPD5,A0A0G2JS76,A0A024R9Y1, P10636,A0A024RA19,I3L170,A0A0G2JQJ7,B3KTM0
	5	DNIKHVPGGGSVQIVYKPVDLSKVTSK	28.18	A0A0G2JMX7,A0A024R9Y0,A0A0G2JQJ7,A0A024RA17,A0A0G2JPD5,A0A0G2JS76,A0A024R9Y1,P10636
	5	qEFEVMEDHAGTYGLGDR	27.84	A0A0G2JMX7,A0A024R9Y0,B4DSB3,A0A024RA17,A0A0G2JPD5,A0A0G2JS76,A0A024R9Y1,P10636, A0A024RA19,A0A0G2JQJ7,I3L2Z2
	2	DQGGYTMHQDQEGDTDAGLKESPLQTPTEDGSEEPGSETSDAK	27.71	A0A0G2JMX7,A0A024RA19,A0A0G2JQJ7,A0A024RA17,A0A0G2JPD5,A0A0G2JS76,A0A024R9Y1,P10636
1	5	HLSNVSSTGSIDmVDSPQLATLADEVSASLAKQGL	26.96	A0A0G2JMX7,A0A024R9Y0,B4DSE3,B4DSB3,A0A024RA17,A0A0G2JPD5,A0A0G2JS76,A0A024R9Y1, P10636,A0A024RA19,I3L170,A0A0G2JQJ7,B3KTM0
	42	TDHGAEIVYKSPVVSGDTSPR	26.04	A0A0G2JMX7,A0A024R9Y0,B4DSE3,B4DSB3,A0A024RA17,A0A0G2JPD5,A0A0G2JS76,A0A024R9Y1, P10636,A0A024RA19,I3L170,A0A0G2JQJ7,B3KTM0
	10	QEFEVMEDHAGTYGLGDR	25.09	A0A0G2JMX7,A0A024R9Y0,B4DSB3,A0A024RA17,A0A0G2JPD5,A0A0G2JS76,A0A024R9Y1,P10636, A0A024RA19,A0A0G2JQJ7,I3L2Z2
	2	ESPLQTPTEDGSEEPGSETSDAKSTPTAEDVTAPLVDEGAPGK	25.03	A0A0G2JMX7,A0A0G2JQJ7,A0A024RA17,A0A0G2JPD5,A0A0G2JS76,A0A024R9Y1,P10636
	15	KDQGGYTmHQDQEGDTDAGLKESPLQTPTEDGSEEPGSETSDAK	24.66	A0A0G2JMX7,A0A024RA19,A0A0G2JQJ7,A0A024RA17,A0A0G2JPD5,A0A0G2JS76,A0A024R9Y1,P10636
	4	LQTAPVPMPDLKNVKSKIGSTENLK	24.49	A0A0G2JMX7,A0A024R9Y0,B4DSB3,A0A024RA17,A0A0G2JPD5,A0A0G2JS76,A0A024R9Y1,P10636, A0A024RA19,I3L170,A0A0G2JQJ7,B3KTM0
	3	qEFEVmEDHAGTYGLGDR	23.7	A0A0G2JMX7,A0A024R9Y0,B4DSB3,A0A024RA17,A0A0G2JPD5,A0A0G2JS76,A0A024R9Y1,P10636, A0A024RA19,A0A0G2JQJ7,I3L2Z2
	1	STPTAEDVTAPLVDEGAPGKQAAAQPHTEIPEGTTAEEAGIGDTPSL EDEAAGHVTQA	23.63	A0A024RA17
	6	ESPLQTPTEDGSEEPGSETSDAK	23.15	A0A0G2JMX7,A0A024RA19,A0A0G2JQJ7,A0A024RA17,A0A0G2JPD5,A0A0G2JS76,A0A024R9Y1,P10636
	3	IGSLDNITHVPGGGNK	22.88	A0A0G2JMX7,A0A024R9Y0,B4DSE3,B4DSB3,A0A024RA17,A0A0G2JPD5,A0A0G2JS76,A0A024R9Y1, P10636,A0A024RA19,I3L170,A0A0G2JQJ7,B3KTM0
	2	AAQPHTEIPEGTTAEEAGIGDTPSLEDEAAGHVTQAR	22.1	A0A024RA17
	8	VQSKIGSLDNITHVPGGGNK	22.04	A0A0G2JMX7,A0A024R9Y0,B4DSE3,B4DSB3,A0A024RA17,A0A0G2JPD5,A0A0G2JS76,A0A024R9Y1, P10636,A0A024RA19,I3L170,A0A0G2JQJ7,B3KTM0
	9	QEFEVmEDHAGTYGLGDR	21.6	A0A0G2JMX7,A0A024R9Y0,B4DSB3,A0A024RA17,A0A0G2JPD5,A0A0G2JS76,A0A024R9Y1,P10636, A0A024RA19,A0A0G2JQJ7,I3L2Z2
	12	HVPGGGSVQIVYKPVDLSKVTSK	21.34	A0A0G2JMX7,A0A024R9Y0,A0A0G2JQJ7,A0A024RA17,A0A0G2JPD5,A0A0G2JS76,A0A024R9Y1,P10636
	8	LQTAPVPMPDLKNVKSK	21.24	A0A0G2JMX7,A0A024R9Y0,B4DSB3,A0A024RA17,A0A0G2JPD5,A0A0G2JS76,A0A024R9Y1,P10636, A0A024RA19,I3L170,A0A0G2JQJ7,B3KTM0
	19	TPPAPKTPPSSGEPPKSGDR	21.06	A0A024R9Y0,A0A024RA17,A0A0G2JPD5,A0A024R9Y1,P10636,A0A024RA19,I3L170,A0A0G2JQJ7,B3KTM0
	8	SGYSSPGSPGTPGSR	20.52	A0A0G2JMX7,A0A024R9Y0,A0A024RA17,A0A0G2JPD5,A0A0G2JS76,A0A024R9Y1,P10636,A0A024RA19, I3L170,A0A0G2JQJ7,B3KTM0
	10	DNIKHVPGGGSVQIVYKPVDLSK	19.35	A0A0G2JMX7,A0A024R9Y0,A0A0G2JQJ7,A0A024RA17,A0A0G2JPD5,A0A0G2JS76,A0A024R9Y1,P10636
	9	STPTAEDVTAPLVDEGAPGK	18.7	A0A0G2JMX7,A0A0G2JQJ7,A0A024RA17,A0A0G2JPD5,A0A0G2JS76,A0A024R9Y1,P10636
	4	LQTAPVPMPDLKNVK	18.63	A0A0G2JMX7,A0A024R9Y0,B4DSB3,A0A024RA17,A0A0G2JPD5,A0A0G2JS76,A0A024R9Y1,P10636, A0A024RA19,I3L170,A0A0G2JQJ7,B3KTM0
	9	LQTAPVPmPDLKNVK	18.29	A0A0G2JMX7,A0A024R9Y0,B4DSB3,A0A024RA17,A0A0G2JPD5,A0A0G2JS76,A0A024R9Y1,P10636, A0A024RA19,I3L170,A0A0G2JQJ7,B3KTM0
	13	HVPGGGSVQIVYKPVDLSK	18.19	A0A0G2JMX7,A0A024R9Y0,A0A0G2JQJ7,A0A024RA17,A0A0G2JPD5,A0A0G2JS76,A0A024R9Y1,P10636
	3	LQTAPVPmPDLKNVKSKIGSTENLK	17.97	A0A0G2JMX7,A0A024R9Y0,B4DSB3,A0A024RA17,A0A0G2JPD5,A0A0G2JS76,A0A024R9Y1,P10636, A0A024RA19,I3L170,A0A0G2JQJ7,B3KTM0
1	1	SRLQTAPVPMPDLKNVK	17.81	A0A0G2JMX7,A0A024R9Y0,B4DSB3,A0A024RA17,A0A0G2JPD5,A0A0G2JS76,A0A024R9Y1,P10636, A0A024RA19,I3L170,A0A0G2JQJ7,B3KTM0
	13	VQIINKKLDLSNVQSK	16.51	A0A0G2JMX7,A0A024R9Y0,A0A0G2JQJ7,A0A024RA17,A0A0G2JPD5,A0A0G2JS76,A0A024R9Y1,P10636
	15	AKTDHGAEIVYKSPVVSGDTSPR	15.89	A0A0G2JMX7,A0A024R9Y0,B4DSE3,B4DSB3,A0A024RA17,A0A0G2JPD5,A0A0G2JS76,A0A024R9Y1, P10636,A0A024RA19,I3L170,A0A0G2JQJ7,B3KTM0
	8	TPSLPTPPTR	15.76	A0A0G2JMX7,A0A024R9Y0,A0A024RA17,A0A0G2JPD5,A0A0G2JS76,A0A024R9Y1,P10636,A0A024RA19, I3L170,A0A0G2JQJ7,B3KTM0
	1	LDLSNVQSK	14.89	A0A0G2JMX7,A0A024R9Y0,A0A0G2JQJ7,A0A024RA17,A0A0G2JPD5,A0A0G2JS76,A0A024R9Y1,P10636
1	21	SRTPSLPTPPTR	14.25	A0A0G2JMX7,A0A024R9Y0,A0A024RA17,A0A0G2JPD5,A0A0G2JS76,A0A024R9Y1,P10636, A0A024RA19,I3L170,A0A0G2JQJ7,B3KTM0
	2	NVSSTGSIDMVDSPQLATLADEVSASLAK	13.84	A0A0G2JMX7,A0A024R9Y0,B4DSE3,B4DSB3,A0A024RA17,A0A0G2JPD5,A0A0G2JS76,A0A024R9Y1, P10636,A0A024RA19,I3L170,A0A0G2JQJ7,B3KTM0
	1	LSNVSSTGSIDMVDSPQLATLADEVSASLAK	13.58	A0A0G2JMX7,A0A024R9Y0,B4DSE3,B4DSB3,A0A024RA17,A0A0G2JPD5,A0A0G2JS76,A0A024R9Y1, P10636,A0A024RA19,I3L170,A0A0G2JQJ7,B3KTM0
	5	SPVVSGDTSPR	11.9	A0A0G2JMX7,A0A024R9Y0,B4DSE3,B4DSB3,A0A024RA17,A0A0G2JPD5,A0A0G2JS76,A0A024R9Y1, P10636,A0A024RA19,I3L170,A0A0G2JQJ7,B3KTM0
	2	LQTAPVPmPDLK	10.34	A0A0G2JMX7,A0A024R9Y0,B4DSB3,A0A024RA17,A0A0G2JPD5,A0A0G2JS76,A0A024R9Y1,P10636, A0A024RA19,I3L170,A0A0G2JQJ7,B3KTM0
	2	IGSLDNITHVPGGGNKKIETHKLTFR	9.66	A0A0G2JMX7,A0A024R9Y0,B4DSE3,B4DSB3,A0A024RA17,A0A0G2JPD5,A0A0G2JS76,A0A024R9Y1, P10636,A0A024RA19,I3L170,A0A0G2JQJ7,B3KTM0
	1	qAAAQPHTEIPEGTTAEEAGIGDTPSLEDEAAGHVTQARMVSK	9.43	A0A024RA17
	14	ENAKAKTDHGAEIVYKSPVVSGDTSPR	9.42	A0A0G2JMX7,A0A024R9Y0,B4DSE3,B4DSB3,A0A024RA17,A0A0G2JPD5,A0A0G2JS76,A0A024R9Y1, P10636,A0A024RA19,I3L170,A0A0G2JQJ7,B3KTM0
	2	GADGKTKIATPR	8.91	A0A0G2JMX7,A0A024R9Y0,A0A024RA17,A0A0G2JPD5,A0A0G2JS76,A0A024R9Y1,P10636,A0A024RA19, I3L170,A0A0G2JQJ7,B3KTM0
	27	GAAPPGQKGQANATR	8.9	A0A0G2JMX7,A0A024R9Y0,A0A024RA17,A0A0G2JPD5,A0A0G2JS76,A0A024R9Y1,P10636,A0A024RA19, I3L170,A0A0G2JQJ7
	1	HLSNVSSTGSIDMVDSPQLATLADEVSASLA	8.7	A0A0G2JMX7,A0A024R9Y0,B4DSE3,B4DSB3,A0A024RA17,A0A0G2JPD5,A0A0G2JS76,A0A024R9Y1, P10636,A0A024RA19,I3L170,A0A0G2JQJ7,B3KTM0
	1	VQSKIGSLDNITHVPGGGNKK	8.24	A0A0G2JMX7,A0A024R9Y0,B4DSE3,B4DSB3,A0A024RA17,A0A0G2JPD5,A0A0G2JS76,A0A024R9Y1, P10636,A0A024RA19,I3L170,A0A0G2JQJ7,B3KTM0
1	3	LQTAPVPMPDLK	7.98	A0A0G2JMX7,A0A024R9Y0,B4DSB3,A0A024RA17,A0A0G2JPD5,A0A0G2JS76,A0A024R9Y1,P10636, A0A024RA19,I3L170,A0A0G2JQJ7,B3KTM0
	1	SNVSSTGSIDMVDSPQLATLADEVSASLAK	7.91	A0A0G2JMX7,A0A024R9Y0,B4DSE3,B4DSB3,A0A024RA17,A0A0G2JPD5,A0A0G2JS76,A0A024R9Y1, P10636,A0A024RA19,I3L170,A0A0G2JQJ7,B3KTM0
	4	IPAKTPPAPKTPPSSGEPPKSGDR	7.86	A0A024R9Y0,A0A024RA17,A0A0G2JPD5,A0A024R9Y1,P10636,A0A024RA19,I3L170,A0A0G2JQJ7,B3KTM0
	1	LQTAPVPMPDLKNVKSKIGSTENLKHQPGGGK	7.6	A0A0G2JMX7,A0A024R9Y0,B4DSB3,A0A024RA17,A0A0G2JPD5,A0A0G2JS76,A0A024R9Y1,P10636, A0A024RA19,I3L170,A0A0G2JQJ7,B3KTM0
	8	TPPAPKTPPSSGEPPK	7.1	A0A024R9Y0,A0A024RA17,A0A0G2JPD5,A0A024R9Y1,P10636,A0A024RA19,I3L170,A0A0G2JQJ7,B3KTM0
	4	TPPSSGEPPKSGDR	6.52	A0A024R9Y0,A0A024RA17,A0A0G2JPD5,A0A024R9Y1,P10636,A0A024RA19,I3L170,A0A0G2JQJ7,B3KTM0
	1	HLSNVSSTGSIDMVDSP	5.94	A0A0G2JMX7,A0A024R9Y0,B4DSE3,B4DSB3,A0A024RA17,A0A0G2JPD5,A0A0G2JS76,A0A024R9Y1, P10636,A0A024RA19,I3L170,A0A0G2JQJ7,B3KTM0
	1	STGSIDMVDSPQLATLADEVSASLAK	5.79	A0A0G2JMX7,A0A024R9Y0,B4DSE3,B4DSB3,A0A024RA17,A0A0G2JPD5,A0A0G2JS76,A0A024R9Y1, P10636,A0A024RA19,I3L170,A0A0G2JQJ7,B3KTM0
	1	HQPGGGKVQIINKKLDLSNVQSK	5.5	A0A0G2JMX7,A0A024R9Y0,A0A0G2JQJ7,A0A024RA17,A0A0G2JPD5,A0A0G2JS76,A0A024R9Y1,P10636
	1	LQTAPVPMPDLKN	5.19	A0A0G2JMX7,A0A024R9Y0,B4DSB3,A0A024RA17,A0A0G2JPD5,A0A0G2JS76,A0A024R9Y1,P10636, A0A024RA19,I3L170,A0A0G2JQJ7,B3KTM0
	1	AVVR	4.53	A0A0G2JMX7,A0A024R9Y0,Q96PD2,A0A0G2JPD5,A0A0G2JS76,P10636,Q5T1A1,O75128, Q01813,Q5H9F3,Q8IZY2,Q8NEM2,B4DSB3,A0A024RA17,A0A024R9Y1,Q969Y0,P08237,Q8TEQ6, A0A024RA19,I3L170,Q6L8Q7,A0A0G2JQJ7,A6NC98,B3KTM0
	1	NVSSTGSIDmVDSPQLATLADEVSASLAK	4.11	A0A0G2JMX7,A0A024R9Y0,B4DSE3,B4DSB3,A0A024RA17,A0A0G2JPD5,A0A0G2JS76,A0A024R9Y1, P10636,A0A024RA19,I3L170,A0A0G2JQJ7,B3KTM0
	1	VAVVR	4	A0A0G2JMX7,A0A024R9Y0,B4DSB3,A0A024RA17,A0A0G2JPD5,A0A0G2JS76,A0A024R9Y1,P10636, A0A024RA19,I3L170,A0A0G2JQJ7,B3KTM0

*LC-MS/MS raw data searched against Uniprot Human + *Porphyromonas gingivalis* + decoy sequence databases.

Table S4. Demographic information of patients with CP who donated saliva and subgingival plaque samples. (See Fig. 8 of main text.)

ID	Gender F/M	Age	Race W=White B=Black	Ethnicity H=Hispanic NH=Non-Hispanic	Periodontal Disease Diagnosis	Duration Periodontal Dx	Previous Periodontal Tx	Medical Hx	Current Medications	Relavant Family Hx
CB01	M	56	W	NH	Severe chronic periodontitis	3 years ago	Scaling and Root Planing and Surgery 2013	None reported	None reported	No
CB02	M	59	W	NH	Severe chronic periodontitis	4 years ago	None reported	None reported	None reported	No
CB03	F	79	W	NH	Severe chronic periodontitis	3 years ago	Scaling and root Planing in the past year	Osteoarthritis	Tylenol, as needed	No
CB04	M	61	B	NH	Severe chronic periodontitis	7 years ago	Scaling and root Planing in the past year	Asthma	Albuterol and Flovent inhaler, as needed	No
CB05	M	67	W	NH	Severe chronic periodontitis	13 years ago	Scaling and root Planing and surgical Tx in 2012	None reported	Baby aspirin	No
